# Supplementary figures and images for: Transfer learning-based channel estimation in orthogonal frequency division multiplexing systems using data-nulling superimposed pilots
Source: PLoS One. 2022 May 27;17(5):e0268952. doi: 10.1371/journal.pone.0268952 (PMC9140250; doi:10.1371/journal.pone.0268952)

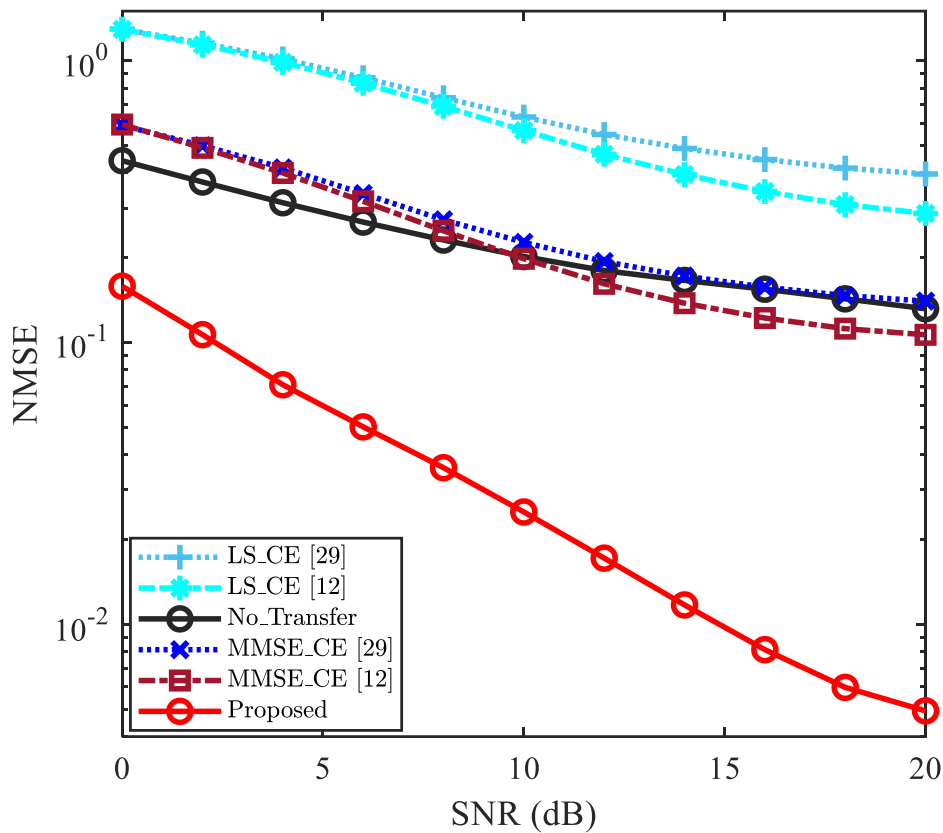

Supplement: S1 Fig — (PDF) [file pone.0268952.s001.pdf]

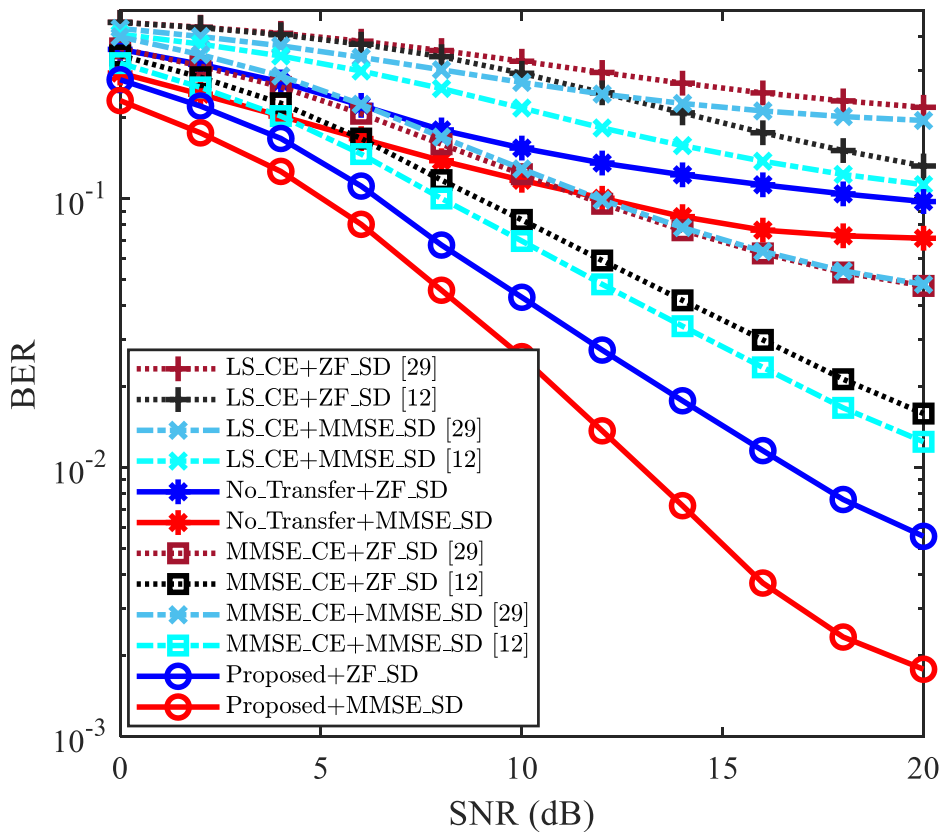

Supplement: S2 Fig — (PDF) [file pone.0268952.s002.pdf]

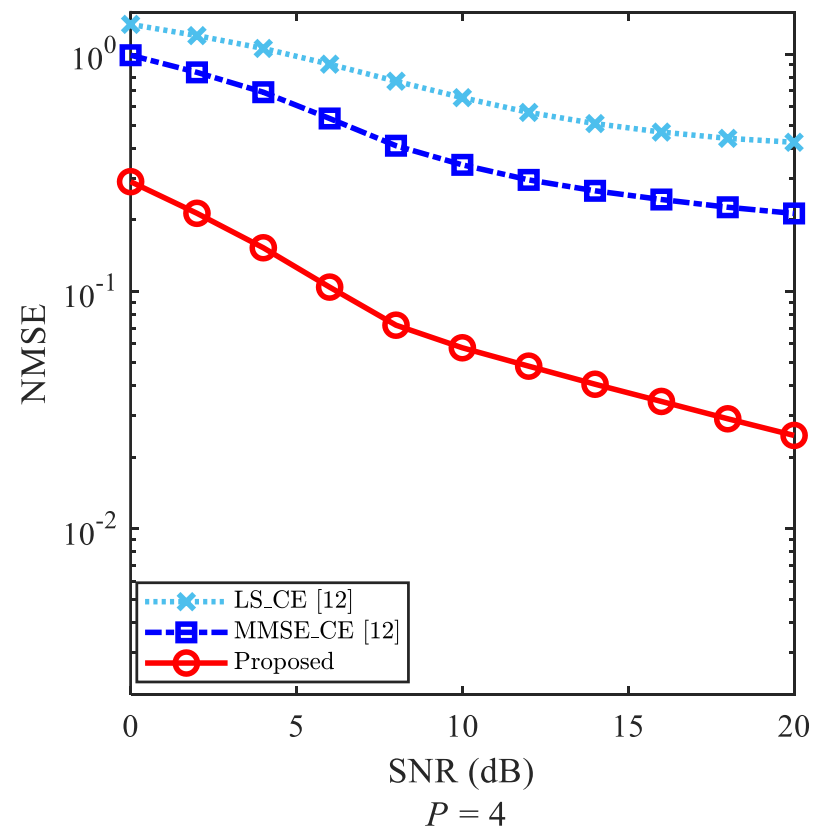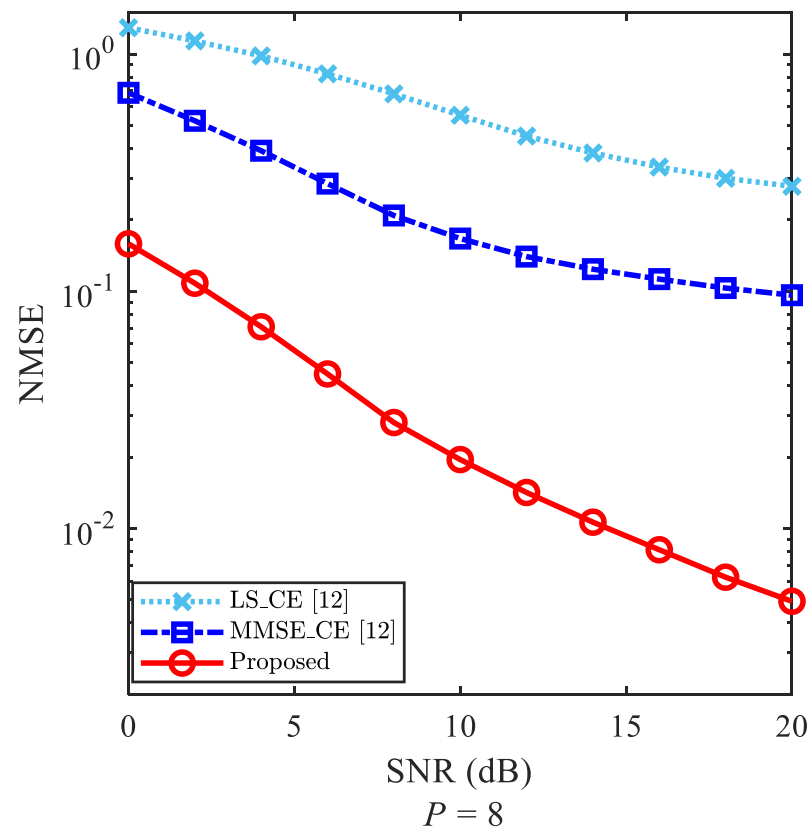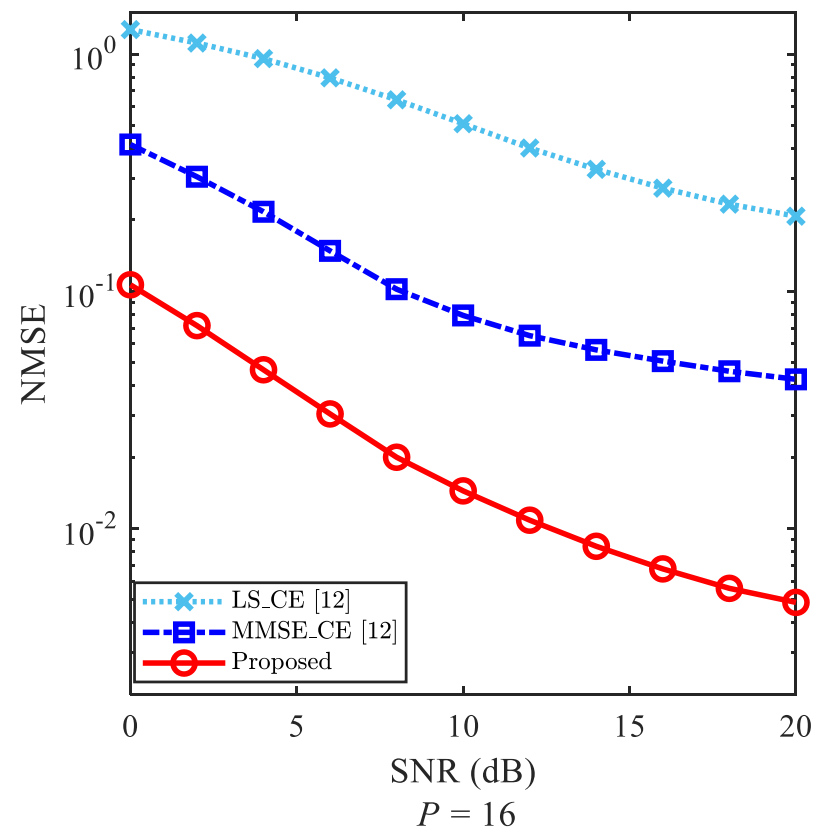

Supplement: S3 Fig — (PDF) [file pone.0268952.s003.pdf]

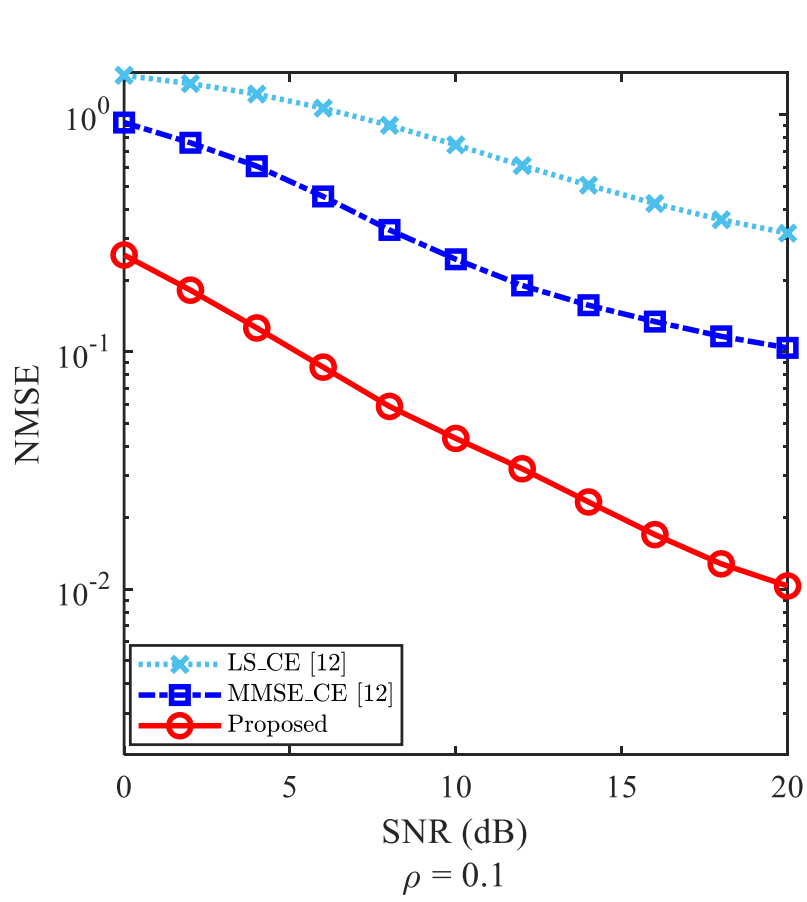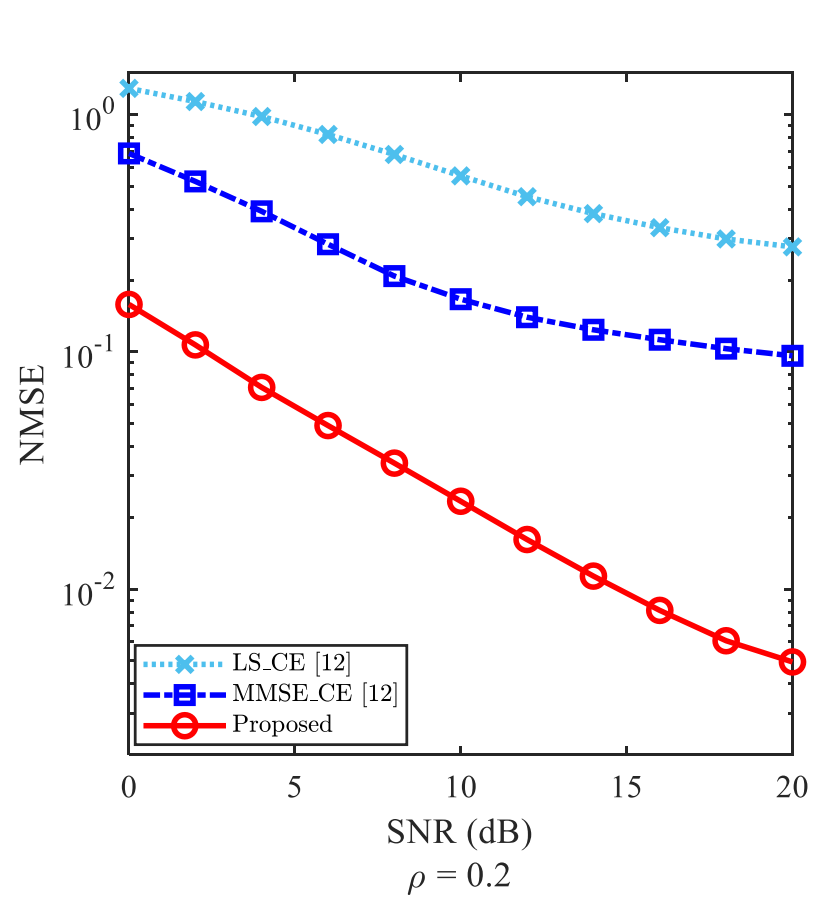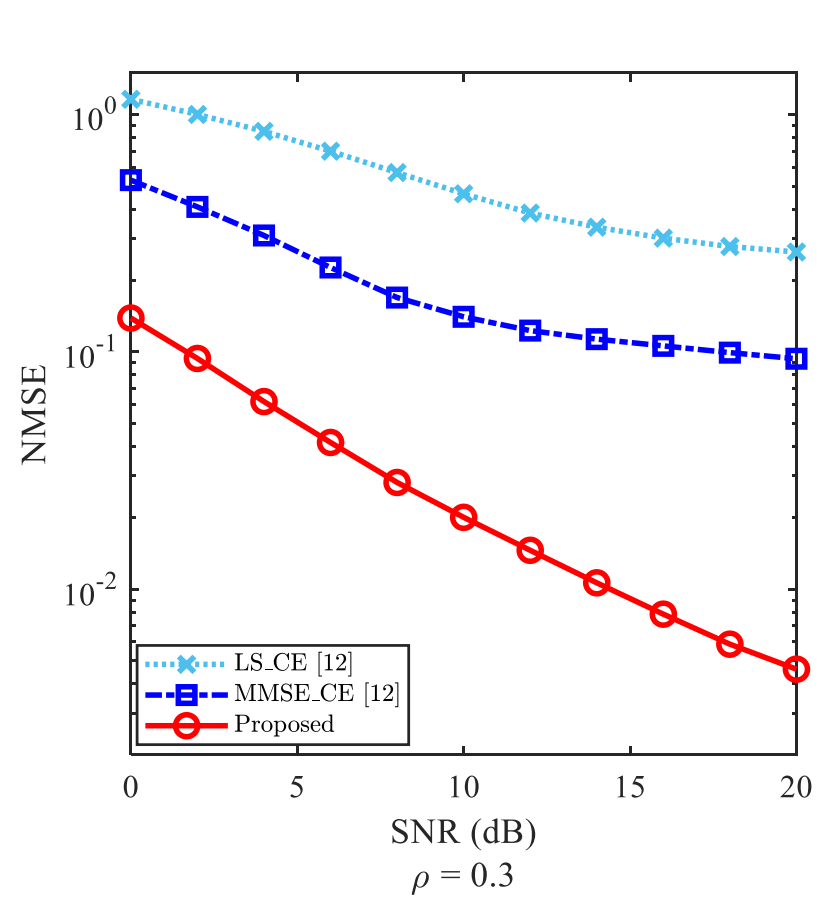

Supplement: S4 Fig — (PDF) [file pone.0268952.s004.pdf]

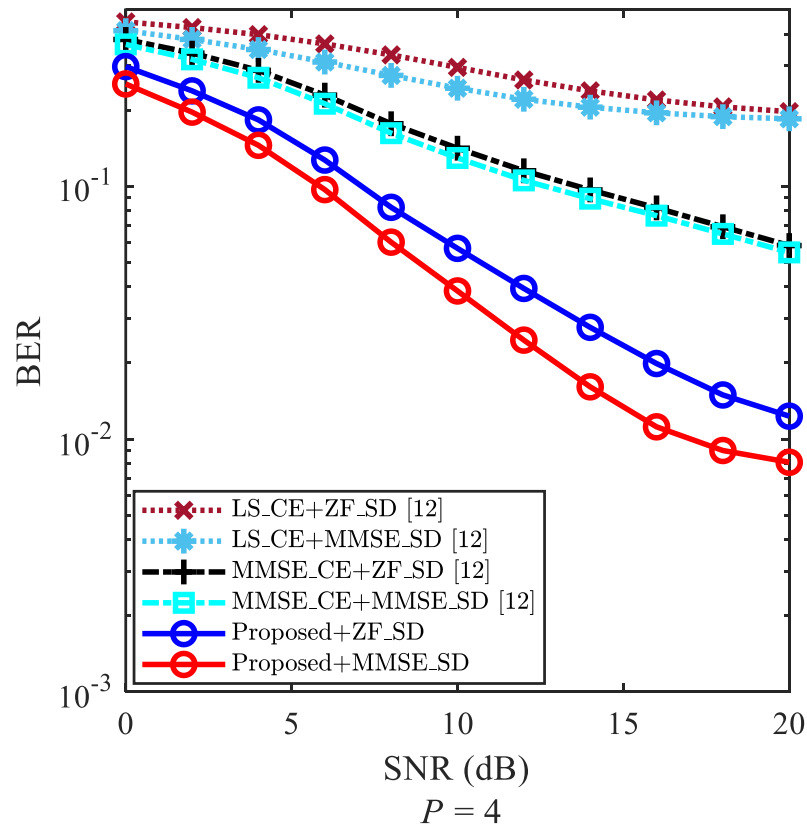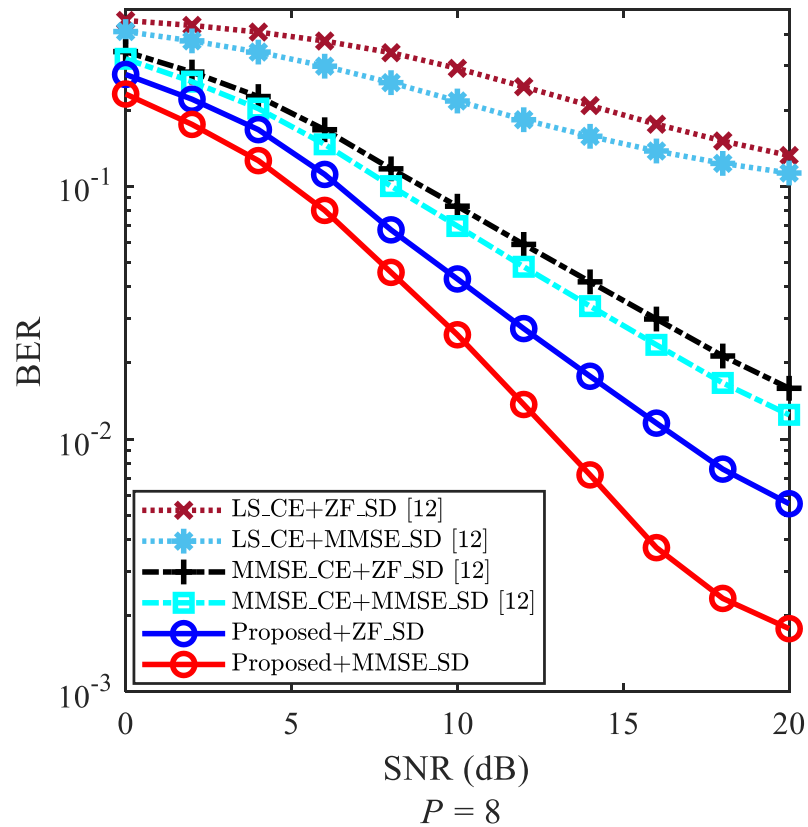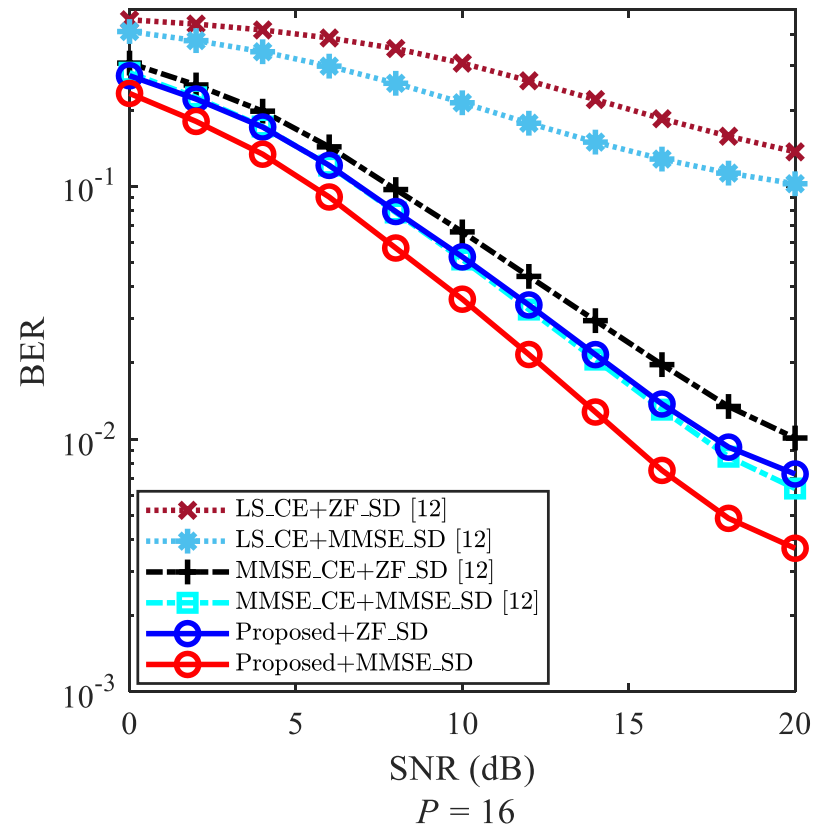

Supplement: S5 Fig — (PDF) [file pone.0268952.s005.pdf]

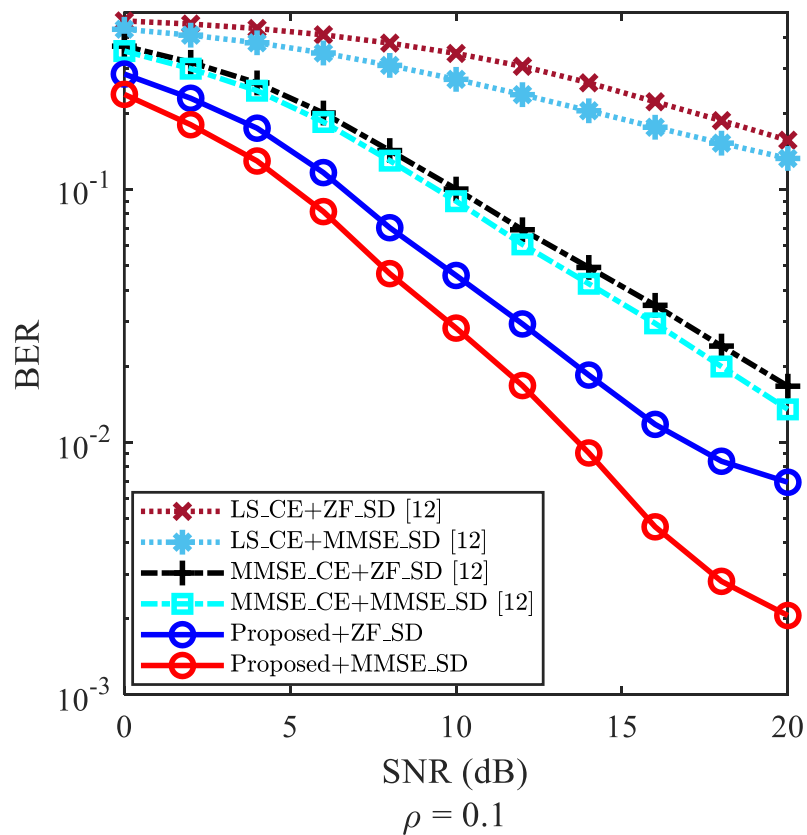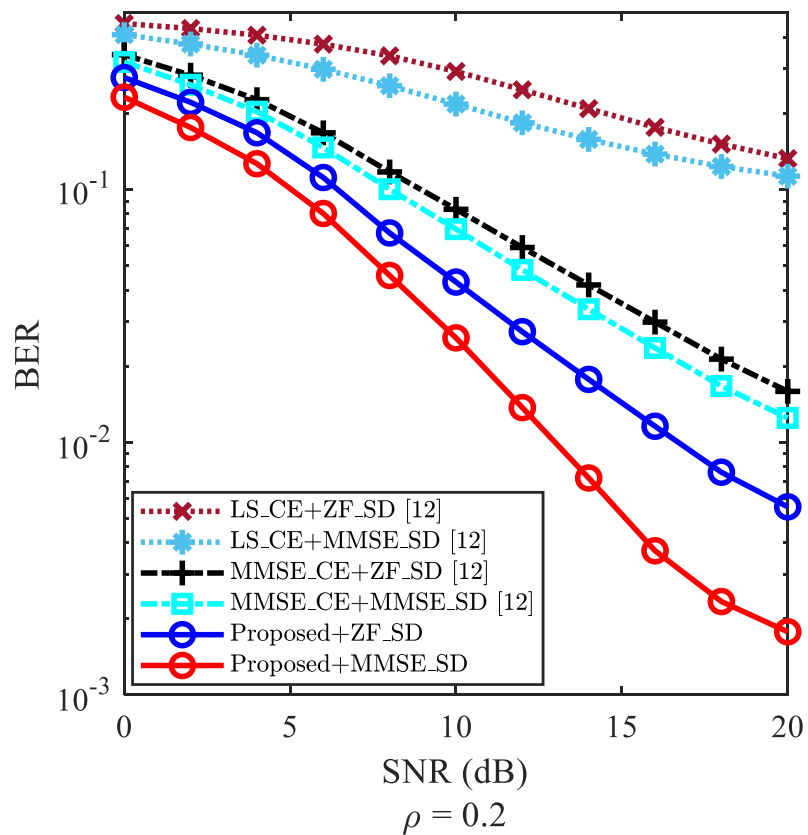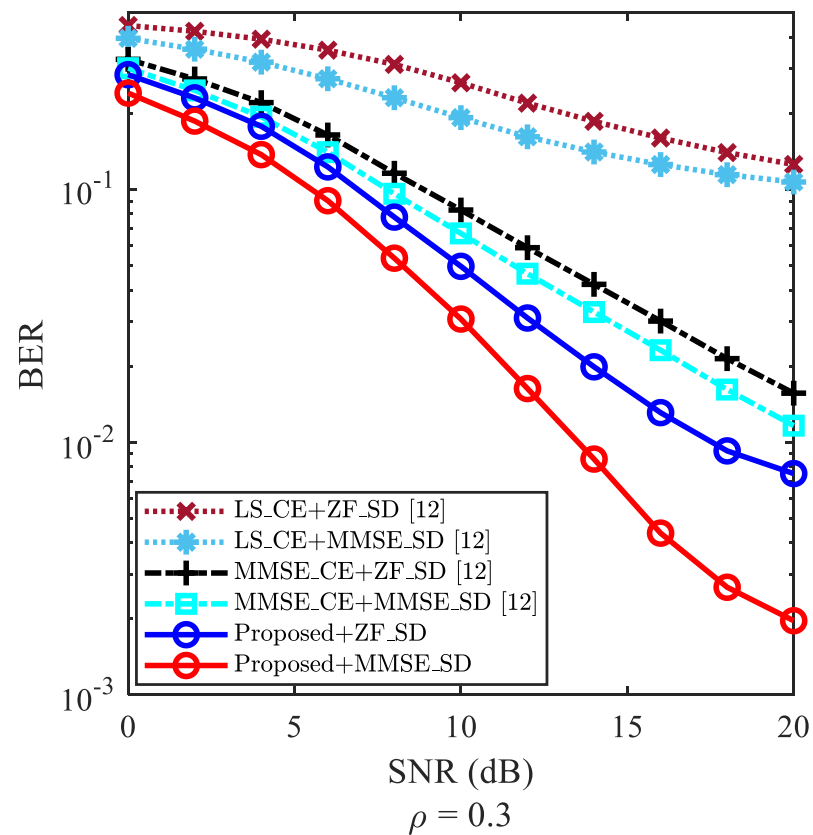

Supplement: S6 Fig — (PDF) [file pone.0268952.s006.pdf]

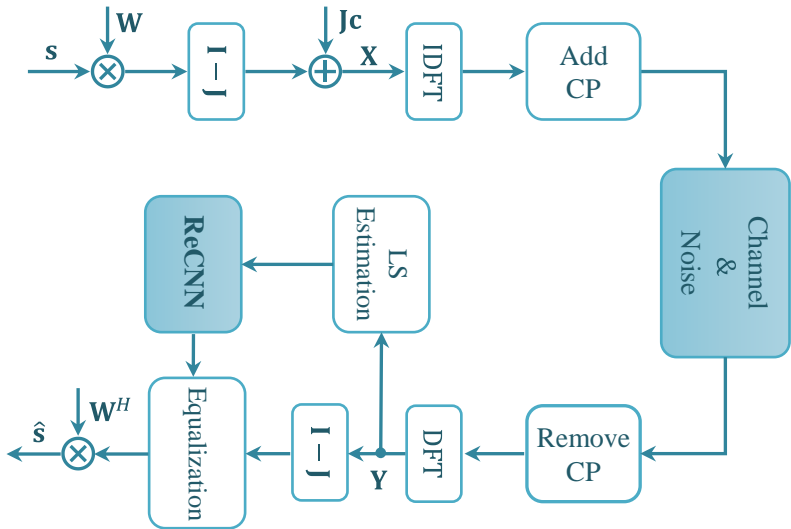

Supplement: S7 Fig — (PDF) [file pone.0268952.s007.pdf]

## Pre-Training Phase

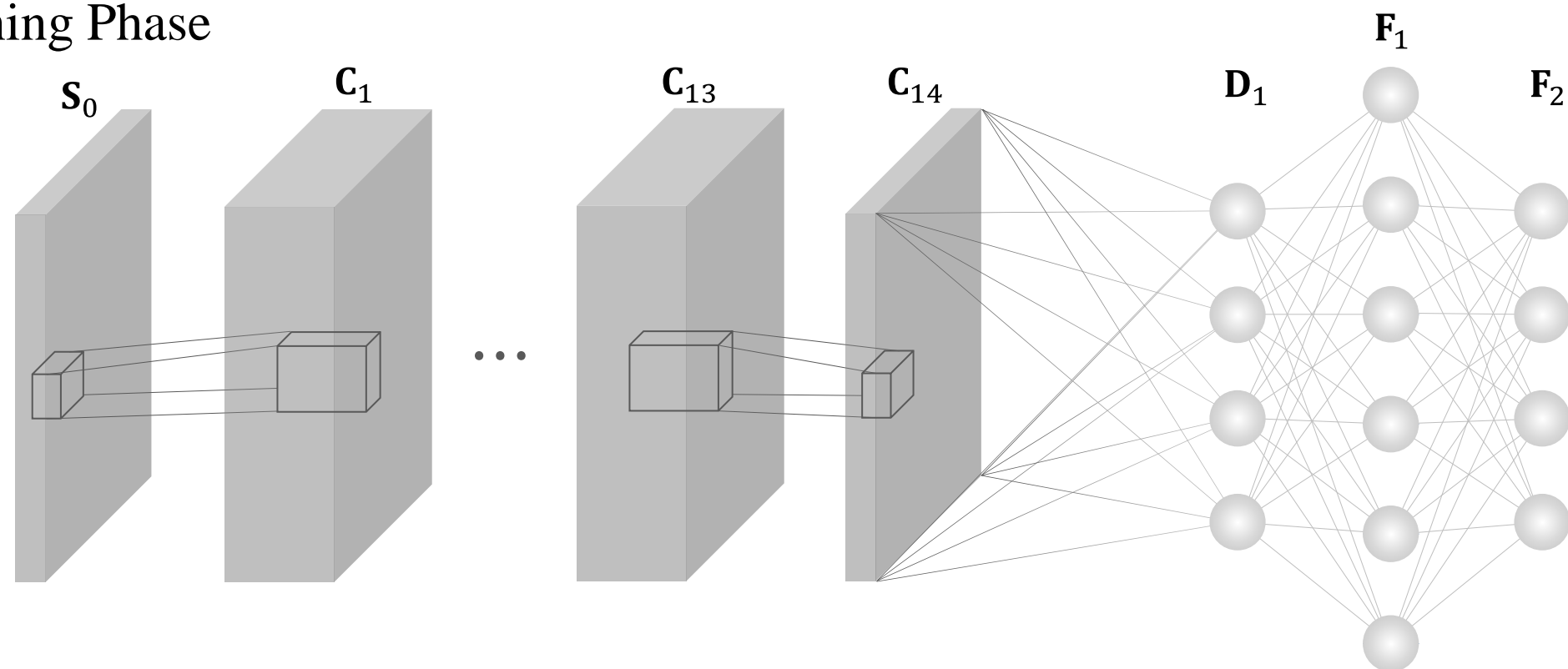

## Testing Phase

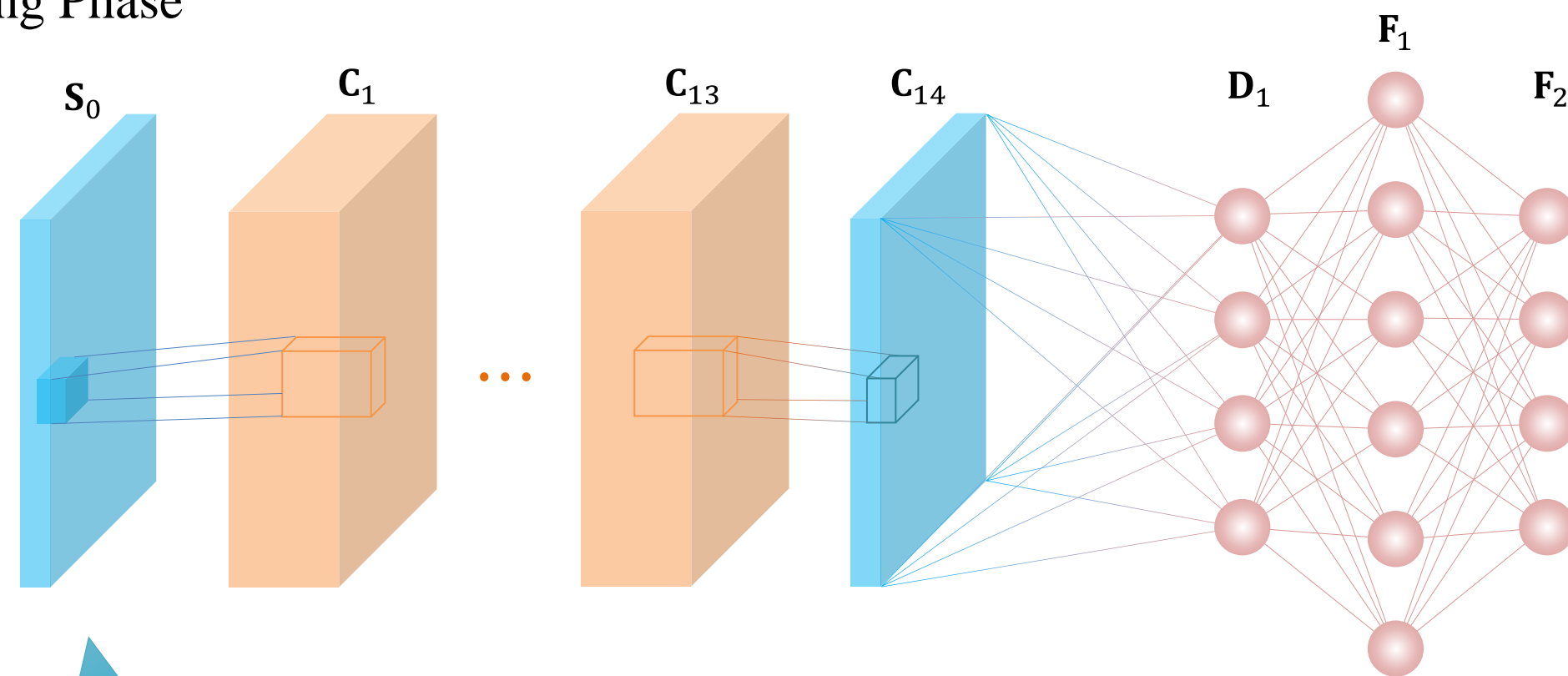

## Fine-Tuning Phase

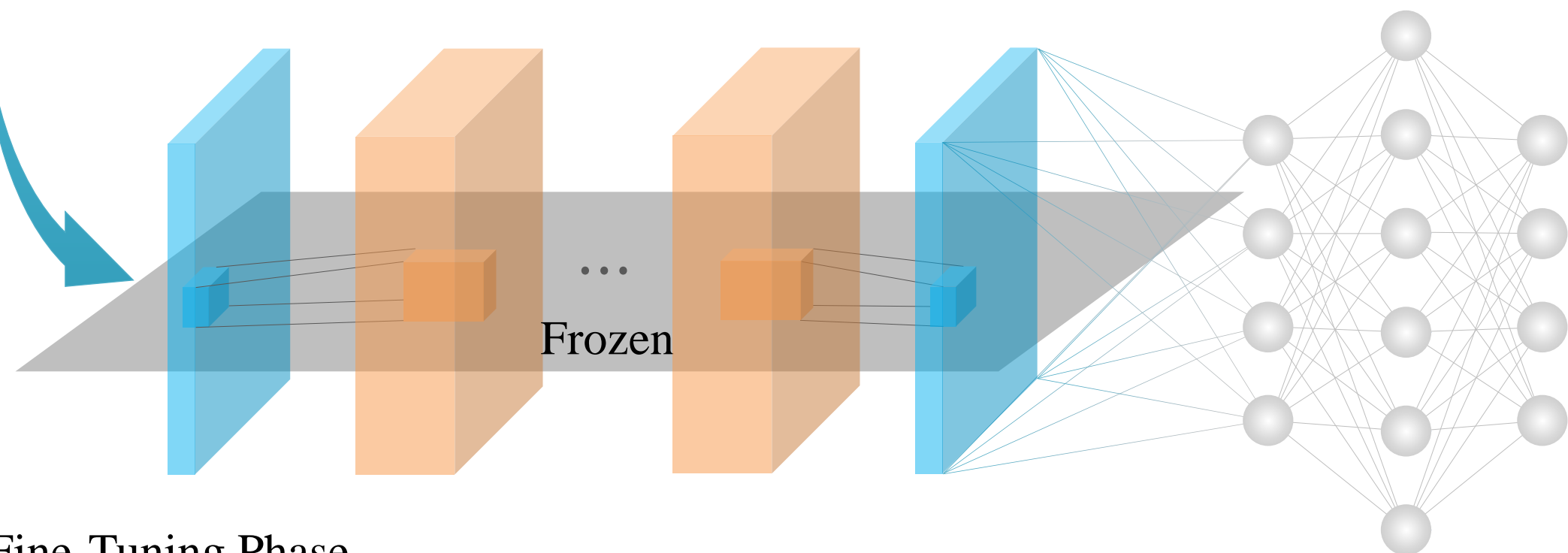

Supplement: S8 Fig — (PDF) [file pone.0268952.s008.pdf]
