# Supplementary material for: Transfer learning-based channel estimation in orthogonal frequency division multiplexing systems using data-nulling superimposed pilots
Source: PLoS One. 2022 May 27;17(5):e0268952. doi: 10.1371/journal.pone.0268952 (PMC9140250; doi:10.1371/journal.pone.0268952)
Supplement: Attachment — Submitted filename: Response to Reviewers.pdf [file pone.0268952.s010.pdf]

Revision of Manuscript ID: [PONE-D-21-33229] Transfer Learning-based Channel Estimation in Orthogonal Frequency Division Multiplexing Systems Using Data-nulling Superimposed Pilots

We would like to express our sincere thanks to the editor and the reviewers for their careful reviews and valuable comments, which have helped us significantly to improve the quality of our work. In the following, we address all comments in detail. All the corresponding changes are highlighted in yellow in the revised manuscript. Please assign this article to the same reviewers.

Reviewer 1:

Comment 1.1: Make the abstract better and clearly state what is the aim/objective of the paper, and what novelty is connected and what experimental results are observed in what $\%$ age and in what parameters.

Response 1.1: Thank you for the comment and suggestion. We apologize for our unclear descriptions. In the revised manuscript, we have modified the abstract and added the experimental results with the recommended form.

Author action: According to the suggestion, we have rewritten the *Abstract* as follows.

"Data-nulling superimposed pilot (DNSP) effectively alleviates the superimposed interference of superimposed training (ST)-based channel estimation (CE) in orthogonal frequency division multiplexing (OFDM) systems, while facing the challenges of the estimation accuracy and computational complexity. By developing the promising solutions of deep learning (DL) in the physical layer of wireless communication, we fuse the DNSP and DL to tackle these challenges in this paper. Nevertheless, due to the changes of wireless scenarios, the model mismatch of DL leads to the performance degradation of CE, and thus faces the issue of network retraining. To address this issue, a lightweight transfer learning (TL) network is further proposed for the DL-based DNSP scheme, and thus structures a TL-based CE in OFDM systems. Specifically, based on the linear receiver, the least squares estimation

is first employed to extract the initial features of CE. With the extracted features, we develop a convolutional neural network (CNN) to fuse the solutions of DL-based CE and the CE of DNSP. Finally, a lightweight TL network is constructed to address the model mismatch. To this end, a novel CE network for the DNSP scheme in OFDM systems is structured, which improves its estimation accuracy and alleviates the model mismatch. The experimental results show that in all signal-to-noise-ratio (SNR) regions, the proposed method achieves lower normalized mean squared error (NMSE) than the existing DNSP schemes with minimum mean square error (MMSE)-based CE. For example, when the SNR is 0 decibel (dB), the proposed scheme achieves similar NMSE as that of the MMSE-based CE scheme at 20 dB, thereby significantly improving the estimation accuracy of CE. In addition, relative to the existing schemes, the improvement of the proposed scheme presents its robustness against the impacts of parameter variations."

Comment 1.2: Add more information to the Introduction section with regard to Problem Definition and Scope of the paper, and even add some more technical highlights covering the related information to the main theme of the paper.

Response 1.2: Thanks for your comments and suggestions. We are deeply sorry for our unclear descriptions in "Introduction". To highlights more technical information of this work, we have rewritten the "Introduction" in the revised manuscript.

Author action: We have rewritten the "Introduction". The Problem Definition and Scope are given in the first three paragraphs of the *Introduction*. We highlighted the motivations of this paper in the fourth paragraph, i.e.,

"Motivated by DNSP, DL, and TL, we fuse the DL-based CE and the DNSP to improve the estimation accuracy and computational complexity, and develop a lightweight TL network to enhance network generalization. To our best knowledge, the TL-based CE for DNSP scheme in OFDM systems has not been investigated. The motivation of this paper is mainly arisen due to the following considerations:

1) Although the ST-based CE saves valuable spectrum resources, its severely superimposed interference needs to be alleviated, triggering us to employ DNSP.
2) The challenges of the estimation accuracy and computational complexity need to be tackled for the CE in DNSP, which promotes us to fuse the DNSP and DL. To this end, the estimation accuracy is improved and the computational complexity is reduced, thereby alleviating the symbol misidentification in DNSP.
3) The developed CE for DNSP scheme should possess the robustness against environmental changes and is not complex. Thus, the dilemmas of network retraining and computational complexity should be solved, impelling us to develop lightweight TL network. With the slightly increased computational complexity, the trained network has a good generalization ability.

".

Comment 1.3: Add Contributions at the end of Introduction. Add Organization of the paper.

Response 1.3: Thanks you for this suggestion. We have added the contributions and organization according to the suggestion in the revised manuscript.

Author action:

(1) In the *Contributions*, on page 4, in addition to modifying the contributions, we have added the following descriptions, i.e.,

1) We develop a framework for fusing DL-based CE and DNSP. To our best knowledge, the DL-based CE for DNSP scheme has not been investigated. We employ DNSP to improve the spectral efficiency, while integrate DL network to enhance estimation accuracy. Our perspective of estimation accuracy improvement is to capture the linear and nonlinear solutions, in which the initial feature for CE is first extracted by the least squares (LS)-based linear estimator followed by a nonlinear DL network. With the developed framework, both the spectral efficiency and estimation accuracy are improved.
2) We introduce the lightweight TL network for DL-based CE with DNSP. From the existing investigations [24], the model mismatch (i.e., the trained network cannot adapt to the change of transmission environment) has not been well addressed by DL-based CE. With the consideration of DNSP scheme, this situation is further worsened. We introduce the lightweight TL network to tackle this issue. Although the lightweight TL network is employed, it substantiality tackles the issue of model mismatch for CE. Especially, the computational complexity and online training time are slightly increased, facilitating its practical applications.
3) We develop a novel ReCNN to improve the estimation accuracy for the CE with DNSP scheme. The developed ReCNN employs CNN to capture a solution of DL-based CE, and utilizes the lightweight TL network to enhance model generalization. Thus, not only the improvement of CE accuracy is achieved by DL-based mode, but also the issue of model mismatch is addressed by TL approach without significant increase of computational complexity. This network architecture provides a good paradigm for the transmission environment adaptability of CE.

(2) In the last paragraph below the *Contributions*, we have added the organization of the revised manuscript, i.e., "The remainder of this paper is structured as follows: In Section II, we describe the system model. The TL-based CE using DNSP method is presented in Section III, followed by the experimental results and analysis are illustrated in Section IV. Finally, Section V concludes our work.".

[24] W. Alves, I. Correa, N. Gonzalez-Prelcic, and A. Klautau, "Deep transfer learning for site-specific channel estimation in low-resolution mmWave MIMO," *IEEE Wireless Commun. Lett.*, vol. 10, no. 7, pp. 1424–1428, Jul. 2021.

Comment 1.4: Under related works, add min 15-25 papers and every paper should be highlighted with what is being proposed, what is the novelty and what experimental results are observed. At the end, highlight

in 9-15 lines what overall technical gaps are observed that led to the design of the proposed methodology.

Response 1.4: Thanks for your comments and suggestions. We are deeply sorry for our inadequate work. We have added 19 papers to describe the *Related Works*. In addition, the technical gaps of our work also have summarized in the last paragraph of *Related Works*.

Author action:

(1) From first paragraph to third paragraph in the *Related Works*, we have added 19 papers to describe the existing investigations, i.e., "In this work, we investigate the TL-based CE for OFDM systems with DNSP. The related works include ST-based CE, DL-based CE, and the TL applications in wireless communication. We respectively review these works as follows.

Relative to ST scheme in [27], DNSP scheme not only improves the CE, but also improves the data detection performance [28]. However, the DNSP discards the partial information of transmitted data symbols, causing the symbol misidentification. In other words, the CE accuracy of DNSP scheme is obtained at the cost of degradation of data detection performance. Thus, the works in [13,29–36] are proposed to tackle this issue. In [13], a partially data-dependent ST scheme was proposed to reduce symbol misidentification by researching the trade-off between interference cancellation and frequency integrity. A partial-data ST was applied to OFDM in [29], in which an interference control factor is assigned for the training sequence. An enhanced scheme of [29] was proposed in [30], which shifted the positions of PS and superimposed them onto the data symbols with the lowest power sum. In [31], authors raised a data detection approach by using an iteration approach with kernel weighted least squares. Due to the increase of computational complexity, the performance of CE and data detection has been improved. [32] investigated a data coding scheme to relieve the symbol misidentification. [33] utilized a constellation rotation scheme to preserve the partial symbol information discarded by the superimposed scheme. By considering the signal sub-space technique, [34] theoretically analyzed that the symbol misidentification was related to both the modulation scheme and the pilot pattern. [35] and [36] respectively investigated the precoding-based approaches which can effectively retrieve the discarded symbol information. Although the symbol misidentification was investigated in [13,29–36], the estimation accuracy and computational complexity of CE remain challenges due to the mode of PS, which has not been well tackled.

Recently, DL-based CE is proposed to improve the estimation accuracy [37]. In [22], an end-to-end approach mode was applied to OFDM system using DL. The DNN network is regarded as a black box for direct signal recovery. [38] constructed a PS designer using two layer neural networks and a channel estimator using DNNs, which were jointly trained to minimize the mean square error (MSE) of CE. Based on [38], the accuracy of CE was further enhanced by using another DNN in an iterative manner [39]. In addition, [40] exploited the DNN to investigate CE for doubly selective fading channels. [41] introduced a residual learning based DNN for CE, in which the computation cost is greatly reduced. Along with the use of DNN, CNN is also commonly used for CE. In [19], two CNNs were utilized to extract the coarse features and refine the CSI accuracy, respectively. [42] introduced a federated learning based framework for downlink channel-CSI prediction, which updates the global model twice by considering the local model weights and the local gradients, respectively. Inspired by CNN, a graph

neural network was constructed in [43] to improve the performance of CE by extracting the correlations of the CSI. Nevertheless, these DL-based CEs still face many challenges, such as low generalization with the environment change [44], long training time, complex parameter tuning, and large memory requirements [45], etc. Relative to the general DL method, the TL features many advantages [24,46], e.g., huge amount of data is not required, the training time is short, and the network effectively adapts to the new environment without network retraining, etc. In [24], the TL approach was exploited to speed up new environment adaptation in low-resolution multiple-input multiple-output systems. By using direct transfer, the TL-based CE was designed in [24] to adapt the migration from one environment to another, while still encountering high computational complexity.

".

(2) At the last paragraph of *Related Works*, the differences between our work and the existing investigations have been added, i.e., "Although these DL-based methods [47] improve the CE accuracy and computational complexity, the DL-based CE for DNSP scheme has not been investigated and faces the challenge of network retraining. The limited TL-based CEs (e.g., [24]) show a good perspective to improve CE generalization. Yet the network architectures need to be lightweight to reduce computational complexity, and thus facilitate their practical deployments. To the best of our knowledge, the TL-based CE for DNSP scheme has not been investigated as well. To remedy the deficiencies of related works (i.e., DNSP, DL and TL) and continue their advantages, we investigate TL-based CE for DNSP scheme in OFDM systems.".

[13] S. He and J. K. Tugnait, "Self-interference suppression in doubly-selective channel estimation using superimposed training," in *Proc. IEEE Int. Conf. Commun.*, Glasgow, UK, June. 2007, pp. 3028–3033.

[19] M. Soltani, V. Pourahmadi, A. Mirzaei, and H. Sheikhzadeh, "Deep learning-based channel estimation," *IEEE Commun. Lett.*, vol. 23, no. 4, pp. 652–655, Apr. 2019.

[22] H. Ye, G. Y. Li, and B. Juang, "Power of deep learning for channel estimation and signal detection in OFDM systems," *IEEE Wireless Commun. Lett.*, vol. 7, no. 1, pp. 114–117, Feb. 2018.

[24] W. Alves, I. Correa, N. Gonzalez-Prelcic, and A. Klautau, "Deep transfer learning for site-specific channel estimation in low-resolution mmWave MIMO," *IEEE Wireless Commun. Lett.*, vol. 10, no. 7, pp. 1424–1428, Jul. 2021.

[28] M. Ghogho, D. McLernon, E. Alameda-Hernandez, and A. Swami, "Channel estimation and symbol detection for block transmission using data-dependent superimposed training," *IEEE Signal Process. Lett.*, vol. 12, no. 3, pp. 226–229, Mar. 2005.

[29] J. C. Estrada-Jimenez and M. J. Fernandez-Getino Garcia, "Partial-data superimposed training with data precoding for OFDM systems," *IEEE Trans. Broadcast.*, vol. 65, no. 2, pp. 234–244, June. 2019.

[30] H. Zhang and B. Sheng, "An enhanced partial-data superimposed training scheme for OFDM systems," *IEEE Commun. Lett.*, vol. 24, no. 8, pp. 1804–1807, May 2020.

[31] C. He, G. Huang, J. Gao, G. Dou, and W. Ying, "Semiblind channel estimation and symbol detection for block transmission using superimposed training," in *Proc. IEEE Int. Conf. Comp. Inf. Technol*, Chengdu, China, Dec. 2012, pp. 627–630.

[32] P. Wang, P. Fan, W. Yuan, and M. Darnell, “Data detection and coding for data-dependent superimposed training,” *IET Signal Process.*, vol. 8, no. 2, pp. 38–145, Apr. 2014.

[33] G. Dou, C. Li, J. Gao, and F. Guo, “Constellation rotation and symbol detection for data-dependent superimposed training,” *Electron. Lett.*, vol. 50, no. 25, pp. 1939–1940, Dec. 2014.

[34] C. Chan, W. Huang, C. Li, and H. Li, “Elimination of data identification problem for data-dependent superimposed training,” *IEEE Trans. Signal Process.*, vol. 63, no. 6, pp. 1595–1604, Mar. 2015.

[35] C. Kuei, C. Li, C. Hung, and W. Huang, “A precoding scheme for eliminating data identification problem in single carrier system using data-dependent superimposed training,” *IEEE Access*, vol. 7, pp. 45930–45939, Apr. 2019.

[36] N. Zhang, N. Cheng, A. T. Gamage, K. Zhang, J. W. Mark, and X. Shen, “Cloud assisted hetnets toward 5G wireless networks,” *IEEE Commun. Mag.*, vol. 53, no. 6, pp. 59–65, June. 2015.

[38] C. Chun, J. Kang, and I. Kim, “Deep learning-based joint pilot design and channel estimation for multiuser MIMO channels,” *IEEE Commun. Lett.*, vol. 23, no. 11, pp. 1999–2003, Nov. 2019

[39] C. Chun, J. Kang, and I. Kim, “Deep learning-based channel estimation for massive mimo systems,” *IEEE Wireless Commun. Lett.*, vol. 8, no. 4, pp. 1228–1231, Aug. 2019.

[40] Y. Yang, F. Gao, X. Ma, and S. Zhang, “Deep learning-based channel estimation for doubly selective fading channels,” *IEEE Access*, vol. 7, pp. 36579–36589, Mar. 2019.

[41] L. Li, H. Chen, H. Chang, and L. Liu, “Deep residual learning meets OFDM channel estimation,” *IEEE Wireless Commun. Lett.*, vol. 9, no. 5, pp. 615–618, May 2020.

[42] Hou W, Sun J, Gui G, Ohtsuki T, Elbir AM, Gacanin H, et al., “Federated Learning for DL-CSI Prediction in FDD Massive MIMO Systems,” *IEEE Wireless Commun. Lett.*, vol. 10, no. 8, pp. 1810–1814, Aug. 2021.

[43] Y.Yang, S.Zhang, F.Gao,J.Ma, and O.A.Dobre, “Graph neural network-based channel tracking for massive MIMO networks,” *IEEE Commun. Lett.*, vol. 24, no. 8, pp. 1747–1751, Aug. 2020.

Comment 1.5: Add flowchart of the proposed methodology.

Response 1.5: Thanks for your comments and suggestions. We have added a flowchart to describe the proposed ReCNN-based TL scheme in Fig 2 of the revised manuscript. In addition, we have added the flowchart of system model in Fig 1 for a better understanding.

Author action:

(1) In the *TL-Based CE*, on page 7, we have added a ReCNN-based TL flowchart, i.e.,

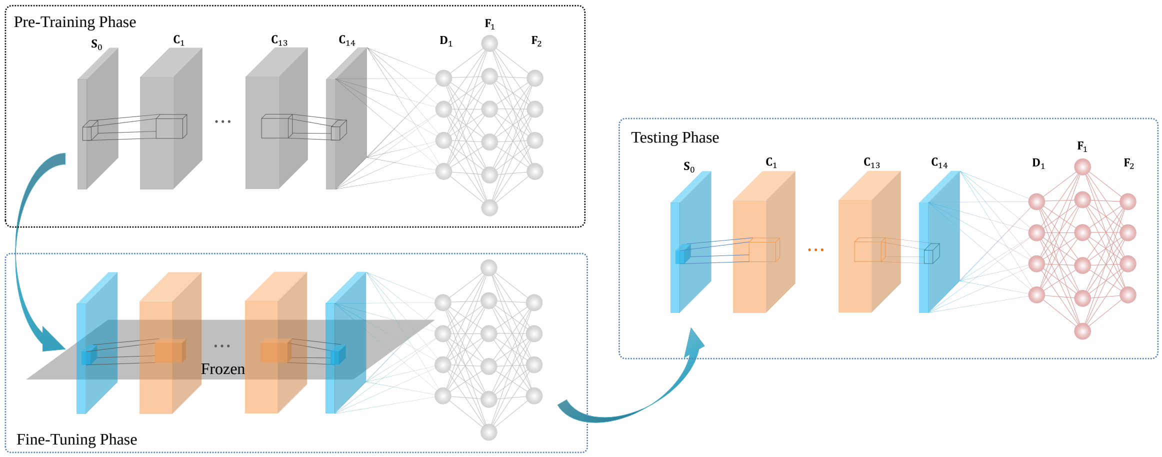

Besides, we have added the corresponding descriptions in the first paragraph of *Model training*, i.e., "Before introducing the ReCNN network training, LS-based CE is first described and then followed by the data collection. At last, the proposed ReCNN-based TL training, i.e., pre-training and fine-tuning are explained. The model training details are summarized in Fig 2".

(2) In the *System Model*, on page 5, we have added a system model flowchart, i.e.,

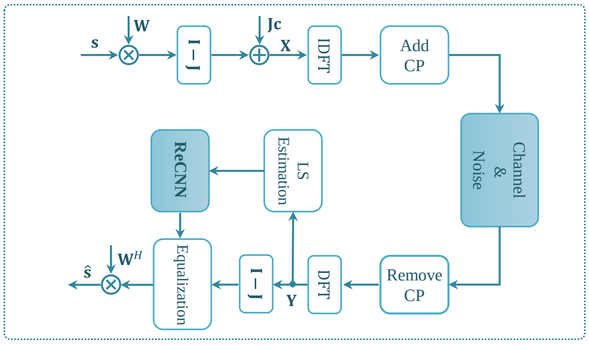

Accordingly, the descriptions of the *System Model* is added in the last paragraph in *System Model*, i.e., "With the received signal $\mathbf{Y}$, the low-complexity LS estimation is first employed to extract the initial features of CE. With the extracted features, a CNN is developed to enhance the CE, and thus structures the framework for fusing DL-based CE and DNSP. Followed by a lightweight TL network, the developed CNN further forms the ReCNN to enhance model generalization. According to the CE produced by ReCNN, we perform the equalization and detection to recovery the transmitted signal. The details of the TL-based CE are elaborated in the next section.".

Comment 1.6: Under experimentation, Add the information of the Dataset.

Response 1.6: Thanks for your comments and suggestions. We have added the information of the Dataset in *Parameter Setting* of the revised manuscript.

Author action: From the fifth to eighth sentences in the first paragraph of *Parameter Setting*, on page 10, we have added the following sentences, i.e., "The data sets $\{\mathbb{D}_{\mathrm{S}}\}$ and $\{\mathbb{D}_{\mathrm{T}}\}$ have 8,000 and 500 samples, respectively. For both of them, the batch sizes are set as 20 samples. In pre-training phase, the data set $\{\mathbb{D}_{\mathrm{S}}\}$ is divided into training set and validation set with the sizes of 6000 and 2000, respectively. Only 500 samples are employed for the $\{\mathbb{D}_{\mathrm{T}}\}$, in which 300 and 200 samples are respectively allocated to $\{\mathbb{D}_{\mathrm{TTr}}\}$ and $\{\mathbb{D}_{\mathrm{TTe}}\}$.".

Comment 1.7: Stress more on the Results and add Tables with Data values on the basis of which graphs are plotted.

Response 1.7: Thank you for the suggestion. In the revised manuscript, we have added Table 2 as a benchmark to present the NMSEs of different methods. Accordingly, additional descriptions are given to explain this Table.

Author action: According to the suggestion, we have added Table 2 in the revised manuscript, i.e.,

SNR / Method	0dB	2dB	4dB	6dB	8dB	10dB	12dB	14dB	16dB	18dB	20dB
LS_CE [29]	1.295	1.154	1.015	0.871	0.739	0.632	0.547	0.488	0.446	0.416	0.396
LS_CE [12]	1.292	1.138	0.986	0.832	0.688	0.565	0.465	0.394	0.343	0.308	0.287
No_Transfer	0.442	0.371	0.313	0.267	0.231	0.202	0.181	0.166	0.154	0.142	0.131
MMSE_CE [29]	0.589	0.499	0.416	0.337	0.272	0.226	0.193	0.172	0.157	0.146	0.141
MMSE_CE [12]	0.593	0.491	0.399	0.317	0.249	0.198	0.161	0.137	0.122	0.112	0.106
**Proposed**	**0.1586**	**0.1067**	**0.0709**	**0.0502**	**0.0359**	**0.0251**	**0.0172**	**0.0117**	**0.0081**	**0.0059**	**0.0049**

Accordingly, the descriptions for Table 2 are added, i.e., "The effectiveness of the NMSE of CE is presented in Fig 3, and partial numerical results are presented in Table 2 for the convenience of comparison." and "Moreover, from Table 2, the NMSE of "No_Transfer" is smaller than that of "MMSE_CE [12]" in the relatively low SNR region (e.g., SNR$\leq$10dB).".

Comment 1.8: Add performance comparison of the proposed work with existing techniques.

Response 1.8: Thank you for the suggestion. In the revised manuscript, we have added the performance comparison of the proposed work with existing techniques in Fig 3 and Fig 4. Accordingly, the descriptions of the added performance comparison have been modified.

Author action:

1) In Fig 3, we have added the performance comparison of NMSE, in which we compare the CE in [12], the CE in [29], and the proposed CE. Accordingly, we have added the descriptions for Fig 3. Please see the sentences from second sentence to third sentence in the second paragraph of *CE and Symbol Detection (SD) Performance*, on page 11, i.e., “From Fig 3, the NMSE of [29] is higher than that of [12]. The reason is the partially data-dependent ST scheme is employed in [29], which superimposes the data sequence on the transmitted PS and thus introduces the superimposed interference into its CE. ...”.

2) Compared with [12] and [29], the BER curves with ZF and MMSE equalizations are given in Fig 4. Correspondingly, its descriptions are added from the fourth sentence to the fifth sentence in the third paragraph of *CE and Symbol Detection (SD) Performance*, on page 13, i.e., “In Fig 4, we compare the BERs among those of [12], [29], and the proposed scheme. With the same CE methods and equalization methods, the BER of [12] is lower than that of [29] due to the influence of CE. ...”.

3) Below equation (11) on page 11, we have modified the simplified expressions, i.e., “

- “Proposed” is used to denote the proposed TL-based CE, “No_Transfer” denotes the proposed scheme without transferring. “LS_CE [12]” and “MMSE_CE [12]” represent “LS channel estimation in [12] ” and “MMSE channel estimation in [12]”, respectively. Meanwhile, “LS_CE [29]” and “MMSE_CE [29]” represent “LS channel estimation in [29] ” and “MMSE channel estimation in [29]”, respectively.
- “LS_CE+ZF_SD [12]”, “LS_CE+MMSE_SD [12]”, “MMSE_CE+ZF_SD [12]”, and “MMSE_CE+MMSE_SD [12]” stand for the “LS channel estimation followed by zero forcing (ZF) equalization in [12]”, “LS channel estimation followed by MMSE equalization in [12]”, “MMSE channel estimation followed by ZF equalization in [12]”, and “MMSE channel estimation followed by MMSE equalization in [12]”, respectively. Meanwhile, “LS_CE+ZF_SD [29]”, “LS_CE+MMSE_SD [29]”, “MMSE_CE+ZF_SD [29]”, and “MMSE_CE+MMSE_SD [29]” stand for the “LS channel estimation followed by ZF equalization in [29]”, “LS channel estimation followed by MMSE equalization in [29]”, “MMSE channel estimation followed by ZF equalization in [29]”, and “MMSE channel estimation followed by MMSE equalization in [29]”, respectively.
- “Proposed+ZF_SD”, “Proposed+MMSE_SD”, “No_Transfer+ZF_SD”, and “No_Transfer+MMSE_SD” stand for the “proposed transfer learning channel estimation followed by ZF equalization”, “proposed transfer learning channel estimation followed by MMSE equalization”, “without transfer learning channel estimation followed by ZF equalization”, and “without transfer learning channel estimation followed by MMSE equalization”, respectively.

[12] G. Dou, C. He, C. Li, and J. Gao,“Channel estimation and symbol detection for OFDM systems using data-nulling superimposed pilots,” *Electron Lett.*, vol. 50, no. 3, pp. 179–180, Jan. 2014.

[29] J. C. Estrada-Jimenez and M. J. Fernandez-Getino Garcia, “Partial-data superimposed training with data precoding for OFDM systems,” *IEEE Trans. Broadcast.*, vol. 65, no. 2, pp. 234–244, June 2019.

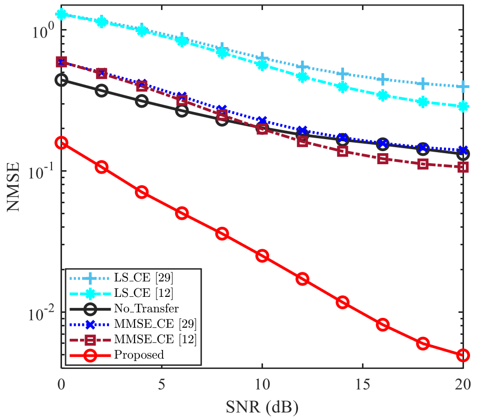

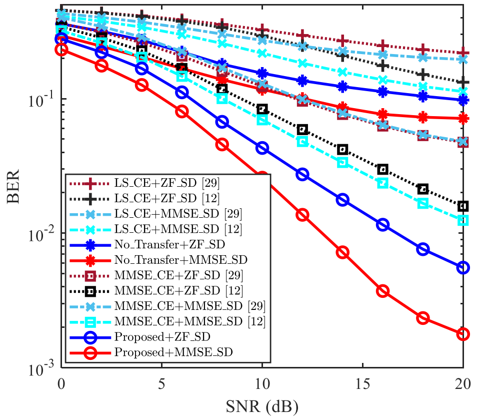

Comment 1.9: Add case study based discussion to the paper.

Response 1.9: Thank you for the suggestion. We have added corresponding discussion in *Analysis of Parameter Impact* of the revised manuscript.

Author action: On page 14 and page 15, we have added the corresponding BER experiments in Fig 6 and Fig 8 to discuss the impacts of P and ρ, respectively. Accordingly, we have added the corresponding descriptions for Fig 6 and Fig 8, respectively. Please see the third paragraph in *Impact of* P and *Impact of* ρ, i.e.,

1) "Fig 6 gives the BER performance with the different equalization methods against the impact of P. From Fig 6, the varying of BER is not regular. The reason is that the performance of SD is affected by both P and the performance of CE. Thus, to achieve a lower BER, the pilot number should be trade off in the proposed scheme. Nevertheless, for each given P, the BERs of "Proposed+ZF_SD" and "Proposed+MMSE_SD" obtain smaller BERs than those of [12] with the same SD methods. This validates that the proposed scheme improves the SD performance and has its robustness against the varying of P."

2) "In Fig 8, the BERs of SD are obtained based on three CE methods, i.e., "Proposed", "LS_CE [12]", and "MMSE_CE [12]". From Fig 8, with the increase of ρ, the BERs of the different methods do not change greatly.

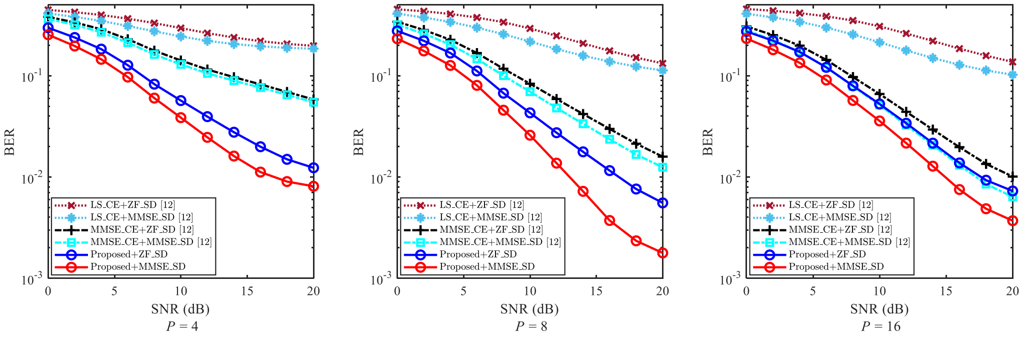

Thus, although the power factor ρ influences the performance of CE, it has a little impact on SD due to the superimposed pilots on the data-nulling in DNSP scheme. Even so, the significant improvement of BER performance is still obtained. For each given ρ, the BERs of "Proposed+ZF_SD" and "Proposed+MMSE_SD" obtain smaller BERs than those of SD methods in [12]. This indicates that the proposed scheme improves the SD performance and has its robustness with the varying of ρ."

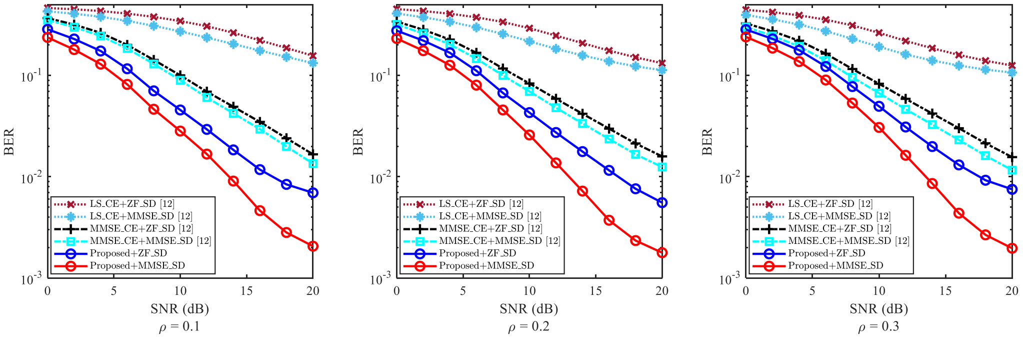

Comment 1.10: Add future scope to this paper.

Response 1.10: Thank you for the suggestion. We have added corresponding future scope in the "Conclusion" of the revised manuscript.

Author action: In the last sentence of the *Conclusion*, on page 16, we have added corresponding future scope, i.e., "In future works, we will investigate the online learning-based CE for the DNSP scheme in OFDM systems.".

Reviewer 2:

Comment 2.1: The abstract should be revised as it does not enough chiefly introduce the area of research along with the research question.

Response 2.1: Thank you for the comment and suggestion. We have rewritten the "Abstract" in the revised manuscript to chiefly introduce the area of research along with the research question.

Author action: In the *Abstract*, we have rewritten the abstract, i.e.,

"Data-nulling superimposed pilot (DNSP) effectively alleviates the superimposed interference of superimposed training (ST)-based channel estimation (CE) in orthogonal frequency division multiplexing (OFDM) systems, while facing the challenges of the estimation accuracy and computational complexity. By developing the promising solutions of deep learning (DL) in the physical layer of wireless communication, we fuse the DNSP and DL to tackle these challenges in this paper. Nevertheless, due to the changes of wireless scenarios, the model mismatch of DL leads to the performance degradation of CE, and thus faces the issue of network retraining. To address this issue, a lightweight transfer learning (TL) network is further proposed for the DL-based DNSP scheme, and thus structures a TL-based CE in OFDM systems. Specifically, based on the linear receiver, the least squares estimation is first employed to extract the initial features of CE. With the extracted features, we develop a convolutional neural network (CNN) to fuse the solutions of DL-based CE and the CE of DNSP. Finally, a lightweight TL network is constructed to address the model mismatch. To this end, a novel CE network for the DNSP scheme in OFDM systems is structured, which improves its estimation accuracy and alleviates the model mismatch. The experimental results show that in all signal-to-noise-ratio (SNR) regions, the proposed method achieves lower normalized mean squared error (NMSE) than the existing DNSP schemes with minimum mean square error (MMSE)-based CE. For example, when the SNR is 0 decibel (dB), the proposed scheme achieves similar NMSE as that of the MMSE-based CE scheme at 20 dB, thereby significantly improving the estimation accuracy of CE. In addition, relative to the existing schemes, the improvement of the proposed scheme presents its robustness against the impacts of parameter variations.".

Comment 2.2: Introduction should be rewritten in a professional way.

Response 2.2: Thank you for this suggestion. We have rewritten the "Introduction" in the revised manuscript.

Author action: The "Introduction" has been rewritten on page 1 to 2, i.e.,

"Orthogonal frequency division multiplexing (OFDM) has been widely applied in wireless communication systems, due to its attractive solution to combat multipath fading [1]. To guarantee the reliable communication in OFDM systems, channel estimation (CE) plays a critical role [2] to eliminate the impact of wireless channels, and thus inspires many CE methods, e.g., non-pilot-aided CE [3,4] and pilot-aided CE [5]. Without employing the pilot sequence (PS), the non-pilot-aided CE saves the valuable bandwidth resources [6]. Yet the high computational complexity hinders its applications [7,8]. With employed PS, the pilot-aided CE achieves the high estimation

accuracy, and thus is favored. However, the PS in pilot-aided CE severely occupies the valuable bandwidth resources [9], degrading the spectral efficiency of OFDM systems. To avoid bandwidth resource occupation, superimposed training (ST)-based CE was proposed. In ST-based CE, the available PS is superimposed on the data sequence [10], and thus saves the bandwidth resources, facilitating its practical applications [11].

Nevertheless, the superimposition between the data sequence and PS introduces the superimposed interference, which seriously affects the performance of CE and subsequent signal detection at the receiver. To suppress the superimposed interference, the variants of ST-based CE are triggered. In these variants, data-nulling superimposed pilot (DNSP) [12] is paid much attention due to its superiority in interference avoidance. The interference between data sequence and PS is avoided by arranging the PS in the frequency bins of data-null [12]. Regretfully, some information-bearing data at certain frequency bins in DNSP are removed prior to transmission, resulting in the symbol misidentification [13]. To tackle the issue of the symbol misidentification, the classic estimation algorithms such as minimum mean square error (MMSE) and maximum likelihood are introduced into DNSP [14]. Yet they are still facing many challenges, such as the availability for the second-order statistics of the channel and noise [15], the low estimation accuracy, and the high computational complexity [16].

To improve the estimation accuracy and reduce the computational complexity, deep learning (DL)-based CE schemes have been proposed in recent years [17]. In [18], two deep neural networks (DNNs) were designed to refine the channel state information (CSI) accuracy and improve the performance of data detection, respectively. Furthermore, convolutional neural network (CNN) is also commonly used in CE [19], which improves the accuracy of CE with reduced computational complexity. However, DL-based CE for OFDM system with DNSP has not been investigated, leaving a huge blank for avoiding the occupation of bandwidth resource. More importantly, these DL-based CE schemes, e.g., [20,21], encounter serious model mismatch. When a network model is trained at one base station but then used at another base station (new environment), the network model usually needs to be retrained [22]. In order to avoid retraining the network model, transfer learning (TL) has been developed in wireless communication systems, e.g, signal detection [23], CE [24] and CSI feed back [25,26], etc. As one of the effective options, TL-based CE is a promising solution for the OFDM systems.".

Comment 2.3: Please explain the proposed method in more details, what is the novelty of the proposed method compared to the state of the art?

Response 2.3: Thank you for the comment and suggestion. We have rewritten the Motivation and Contribution. In the rewritten Motivation and Contribution, we present the novelty of the proposed method compared to the state of the art, i.e., compared with deep learning (DL)-based channel estimation (CE) and superimposed training (ST)-based CE, the DL-based CE for DNSP scheme has not been investigated. We employ DNSP to improve the spectral efficiency, while integrate DL network to enhance estimation accuracy. In order to solve the model mismatch, we

introduce the lightweight transfer learning (TL) network for DL-based CE with DNSP.

Author action:

(1) In the fourth paragraph of the *Introduction*, on page 2, we have rewritten the motivation, i.e.,

"Motivated by DNSP, DL, and TL, we fuse the DL-based CE and the DNSP to improve the estimation accuracy and computational complexity, and develop a lightweight TL network to enhance network generalization. To our best knowledge, the TL-based CE for DNSP scheme in OFDM systems has not been investigated. The motivation of this paper is mainly arisen due to the following considerations:

1) Although the ST-based CE saves valuable spectrum resources, its severely superimposed interference needs to be alleviated, triggering us to employ DNSP.
2) The challenges of the estimation accuracy and computational complexity need to be tackled for the CE in DNSP, which promotes us to fuse the DNSP and DL. To this end, the estimation accuracy is improved and the computational complexity is reduced, thereby alleviating the symbol misidentification in DNSP.
3) The developed CE for DNSP scheme should possess the robustness against environmental changes and is not complex. Thus, the dilemmas of network retraining and computational complexity should be solved, impelling us to develop lightweight TL network. With the slightly increased computational complexity, the trained network has a good generalization ability.

".

(2) In the *Contributions*, on page 4, we have rewritten the contribution, i.e.,

"The main contributions of this paper are summarized as follows.

1) We develop a framework for fusing DL-based CE and DNSP. To our best knowledge, the DL-based CE for DNSP scheme has not been investigated. We employ DNSP to improve the spectral efficiency, while integrate DL network to enhance estimation accuracy. Our perspective of estimation accuracy improvement is to capture the linear and nonlinear solutions, in which the initial feature for CE is first extracted by the least squares (LS)-based linear estimator followed by a nonlinear DL network. With the developed framework, both the spectral efficiency and estimation accuracy are improved.
2) We introduce the lightweight TL network for DL-based CE with DNSP. From the existing investigations [24], the model mismatch (i.e., the trained network cannot adapt to the change of transmission environment) has not been well addressed by DL-based CE. With the consideration of DNSP scheme, this situation is further worsened. We introduce the lightweight TL network to tackle this issue. Although the lightweight TL network is employed, it substantiality tackles the issue of model mismatch for CE. Especially, the computational complexity and online training time is slightly increased, facilitating its practical applications.
3) We develop a novel ReCNN to improve the estimation accuracy for the CE with DNSP scheme. The developed ReCNN employs CNN to capture a solution of DL-based CE, and utilizes the lightweight TL network to enhance model generalization. Thus, not only the improvement of CE accuracy is achieved by DL-based mode, but also the issue of model mismatch is addressed by TL approach without significant

increase of computational complexity. This network architecture provides a good paradigm for the transmission environment adaptability of CE.

".

Comment 2.4: Current experiments are weak and fair comparison with other recent methods is very necessary.

Response 2.4: Thanks for your comment and suggestion. We do agree that current experiments are weak, and have added the comparison. In the revised manuscript, we have added the performance comparison of the proposed work with existing techniques in Fig 3 and Fig 4. Accordingly, the descriptions of the added performance comparison have been modified.

Author action:

1) In Fig 3, we have added the performance comparison of NMSE, in which we compare the CE in [12], the CE in [29], and the proposed CE. Accordingly, we have added the descriptions for Fig 3. Please see the sentences from second sentence to third sentence in the second paragraph of *CE and Symbol Detection (SD) Performance*, on page 11, i.e., "From Fig 3, the NMSE of [29] is higher than that of [12]. The reason is the partially data-dependent ST scheme is employed in [29], which superimposes the data sequence on the transmitted PS and thus introduces the superimposed interference into its CE. ...".

2) Compared with [12] and [29], the BER curves with ZF and MMSE equalizations are given in Fig 4. Correspondingly, its descriptions are added from the fourth sentence to the fifth sentence in the third paragraph of *CE and Symbol Detection (SD) Performance*, on page 13, i.e., "In Fig 4, we compare the BERs among those of [12], [29], and the proposed scheme. With the same CE methods and equalization methods, the BER of [12] is lower than that of [29] due to the influence of CE. ...".

3) Below equation (11) on page 11, we have modified the simplified expressions, i.e., "

- "Proposed" is used to denote the proposed TL-based CE, "No_Transfer" denotes the proposed scheme without transferring. "LS_CE [12]" and "MMSE_CE [12]" represent "LS channel estimation in [12] " and "MMSE channel estimation in [12]", respectively. Meanwhile, "LS_CE [29]" and "MMSE_CE [29]" represent "LS channel estimation in [29] " and "MMSE channel estimation in [29]", respectively.
- "LS_CE+ZF_SD [12]", "LS_CE+MMSE_SD [12]", "MMSE_CE+ZF_SD [12]", and "MMSE_CE+MMSE_SD [12]" stand for the "LS channel estimation followed by zero forcing (ZF) equalization in [12]", "LS channel estimation followed by MMSE equalization in [12]", "MMSE channel estimation followed by ZF equalization in [12]", and "MMSE channel estimation followed by MMSE equalization in [12]", respectively. Meanwhile, "LS_CE+ZF_SD [29]", "LS_CE+MMSE_SD [29]", "MMSE_CE+ZF_SD [29]", and "MMSE_CE+MMSE_SD [29]" stand for the "LS channel estimation followed by ZF equalization in [29]", "LS channel estimation

followed by MMSE equalization in [29]”, “MMSE channel estimation followed by ZF equalization in [29]”, and “MMSE channel estimation followed by MMSE equalization in [29]”, respectively.

- “Proposed+ZF_SD”, “Proposed+MMSE_SD”, “No_Transfer+ZF_SD”, and “No_Transfer+MMSE_SD” stand for the “proposed transfer learning channel estimation followed by ZF equalization”, “proposed transfer learning channel estimation followed by MMSE equalization”, “without transfer learning channel estimation followed by ZF equalization”, and “without transfer learning channel estimation followed by MMSE equalization”, respectively.

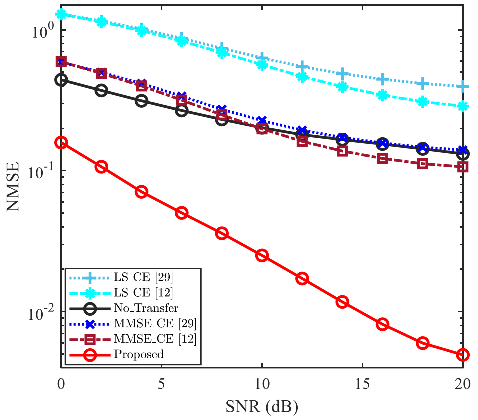

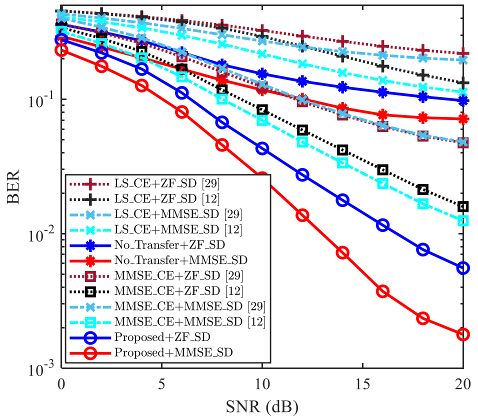

[12] G. Dou, C. He, C. Li, and J. Gao,“Channel estimation and symbol detection for OFDM systems using data-nulling superimposed pilots,” *Electron Lett.*, vol. 50, no. 3, pp. 179–180, Jan. 2014.

[29] J. C. Estrada-Jimenez and M. J. Fernandez-Getino Garcia, “Partial-data superimposed training with data precoding for OFDM systems,” *IEEE Trans. Broadcast.*, vol. 65, no. 2, pp. 234–244, June 2019.

Comment 2.5: The experiment description section is too rough. The description of data collection should be added. For the experimental results, it will be good to present a statistical test in the comparison of the results with other published methods. This can help to support the claim on improved results obtained with the selection methods studied.

Response 2.5: Thanks for your comment and suggestion. We agree that the experiment description section is rough. We have added the description of data collection and added the experimental comparison.

Author action:

1) In the second point of *Model training* on page 8, we have added the descriptions of data collection, i.e.,"In other words, the number of clusters in each environment are randomly distributed rather than fixed.".

2) On page 14 and page 15, we have added the corresponding BER experiments in Fig 6 and Fig 8 to discuss the impacts of P and ρ, respectively. Accordingly, we have added the corresponding descriptions for Fig 6 and Fig 8, respectively. Please see the third paragraph in *Impact of P* and *Impact of* ρ, i.e.,

"Fig 6 gives the BER performance with the different equalization methods against the impact of P. From Fig 6, the varying of BER is not regular. The reason is that the performance of SD is affected by both P and the performance of CE. Thus, to achieve a lower BER, the pilot number should be trade off in the proposed scheme. Nevertheless, for each given P, the BERs of "Proposed+ZF_SD" and "Proposed+MMSE_SD" obtain smaller BERs than those of [12] with the same SD methods. This validates that the proposed scheme improves the SD performance and has its robustness against the varying of P."

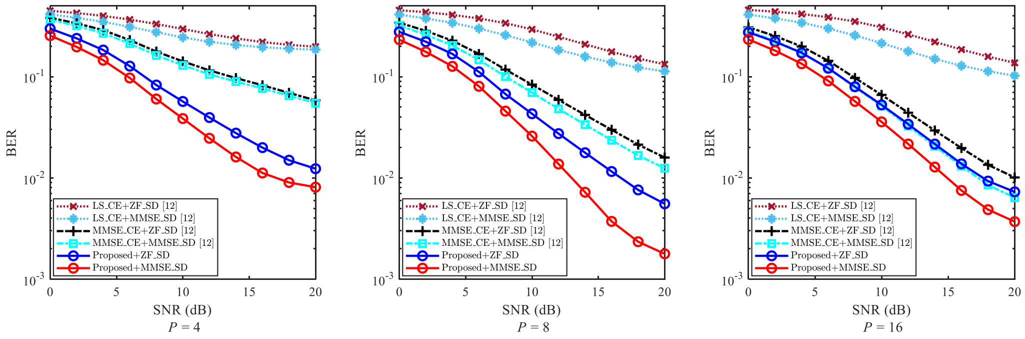

"In Fig 8, the BERs of SD are obtained based on three CE methods, i.e., "Proposed", "LS_CE [12]", and "MMSE_CE [12]". From Fig 8, with the increase of ρ, the BERs of the different methods do not change greatly. Thus, although the power factor ρ influences the performance of CE, it has a little impact on SD due to the superimposed pilots on the data-nulling in DNSP scheme. Even so, the significant improvement of BER performance is still obtained. For each given ρ, the BERs of "Proposed+ZF_SD" and "Proposed+MMSE_SD" obtain smaller BERs than those of SD methods in [12]. This indicates that the proposed scheme improves the SD performance and has its robustness with the varying of ρ."

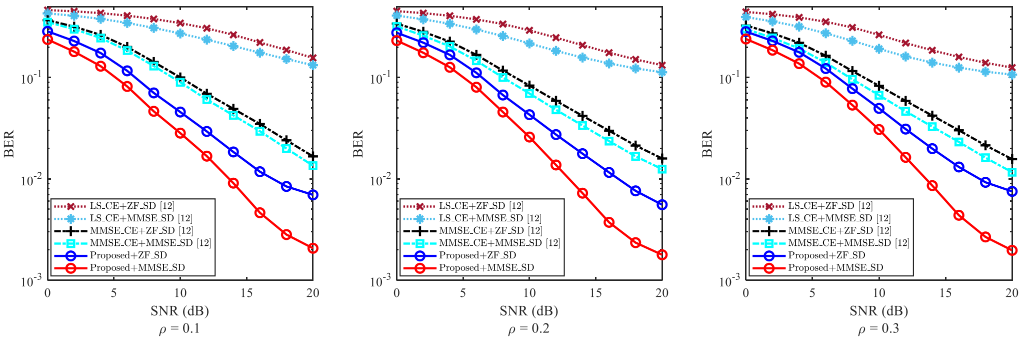

Comment 2.6: Other aspect where paper can be improved is motivation and the reason for the given architecture. The current approach seems to be more like we have this different types of architectures, let's mix them and present results by training them. It would be of great interest why a particular model was selected and what a particular part in the framework is helping us to learn.

Response 2.6: Thanks for your comment and suggestion. We first develop a framework for fusing DL-based CE and the LS estimator for DNSP scheme to improve the spectral efficiency, estimation accuracy and computational complexity. Then, we introduce the lightweight TL network for DL-based CE with DNSP to improve network generalization. To this end, we indeed develop a novel network, i.e., ReCNN. We apologize for our unclear description, and have added corresponding descriptions in the revised manuscript.

Author action: From the first sentence to the second sentence in the first paragraph of *Model Structure*, on page 7, we have added the corresponding descriptions, i.e., "In this paper, we fuse the LS estimator and DL-based CE for the DNSP scheme to capture the non-NN and NN, respectively. To further address the model mismatch of DL-based CE, a lightweight TL network which is inspired by the DnCNN in [19] and named as ReCNN, is proposed in this paper.".

[19] M. Soltani, V. Pourahmadi, A. Mirzaei, and H. Sheikhzadeh, "Deep learning-based channel estimation," *IEEE Commun. Lett.*, vol. 23, no. 4, pp. 652–655, Apr. 2019.

Comment 2.7: English can be improved. Proofreading should ensure appropriate use of grammar, tense and punctuations. Longer sentences should be converted into smaller ones.

Response 2.7: Thanks for your comment and careful reading. We have made a proofreading and corrected the inappropriate grammar, tense and punctuations in the revised manuscript.

Author action: We have made the following corrections in the revised manuscript:

1) In the second sentence of the first paragraph in *System Model*, we have modified "... a unitary matrix ..." as "... an unitary matrix ...".

2) In the first sentence of the *Transfer Learning-based Channel Estimation*, on page 5, we have corrected the "... which caused the symbol misidentification in the receiver." to "... which causes the symbol misidentification at the receiver.".
3) In the first sentence of the *Pre-training*, on page 9, we have modified "Denote the whole network parameters as $\Theta = \{\Theta_{\rm pre}, \Theta_{\rm fin}\}$, with $\Theta_{\rm pre}$ and $\Theta_{\rm fin}$ being the sets of parameter values for *Pre-training* and *Fining-training* phase, respectively." as "We denote the whole network parameters as $\Theta = \{\Theta_{\rm pre}, \Theta_{\rm fin}\}$ of which the $\Theta_{\rm pre}$ and $\Theta_{\rm fin}$ are the sets of parameter values for *Pre-training* and *Fining-training* phase, respectively.".
4) In the sixth sentence of the *Fine-tuning*, on page 10, we have corrected the "... (given in 9) ..." to "(given in (9)) ...".

In addition to the corrections mentioned above, we have corrected other grammar, tense, and sentences in the revised manuscript which have been highlighted.

Reviewer 3:

Comment 3.1: This paper investigates deep transfer NN to solve the issues of channel estimation for DNSP systems. The paper is generally well written, logical and discusses a novel topic.

Response 3.1: Thank you very much for the positive comments on our work.

Comment 3.2: In the abstract, the authors claim that DNSP has high spectrum efficiency. Please elaborate on why?

Response 3.2: Thanks for your comment. The reason why the DNSP has high spectrum efficiency is given as follows. Assuming two OFDM systems, both of them have N subcarriers and P pilots. One of them employs DNSP to superimpose P pilots on N modulated symbols (DNSP-based mode). The other one does not use the superimposition mode (Non-superimposition mode). Then, for each OFDM symbol, N modulated symbols can be transmitted by DNSP-based mode, while only $N-P$ modulated symbols are transmitted by the Non-superimposition mode. Therefore, relative to the Non-superimposition mode, the DNSP mode has higher spectrum efficiency.

Author action: According to the suggestions of the other reviewers, we have rewritten the *Abstract* as follows.

"Data-nulling superimposed pilot (DNSP) effectively alleviates the superimposed interference of superimposed training (ST)-based channel estimation (CE) in orthogonal frequency division multiplexing (OFDM) systems, while facing the challenges of the estimation accuracy and computational complexity. By developing the promising solutions of deep learning (DL) in the physical layer of wireless communication, we fuse the DNSP and DL to tackle these challenges in this paper. Nevertheless, due to the changes of wireless scenarios, the model mismatch of DL leads to the performance degradation of CE, and thus faces the issue of network retraining. To address this

issue, a lightweight transfer learning (TL) network is further proposed for the DL-based DNSP scheme, and thus structures a TL-based CE in OFDM systems. Specifically, based on the linear receiver, the least squares estimation is first employed to extract the initial features of CE. With the extracted features, we develop a convolutional neural network (CNN) to fuse the solutions of DL-based CE and the CE of DNSP. Finally, a lightweight TL network is constructed to address the model mismatch. To this end, a novel CE network for the DNSP scheme in OFDM systems is structured, which improves its estimation accuracy and alleviates the model mismatch. The experimental results show that in all signal-to-noise-ratio (SNR) regions, the proposed method achieves lower normalized mean squared error (NMSE) than the existing DNSP schemes with minimum mean square error (MMSE)-based CE. For example, when the SNR is 0 decibel (dB), the proposed scheme achieves similar NMSE as that of the MMSE-based CE scheme at 20 dB, thereby significantly improving the estimation accuracy of CE. In addition, relative to the existing schemes, the improvement of the proposed scheme presents its robustness against the impacts of parameter variations.".

Comment 3.3: How to design the unitary matrix W in Equation (2). What are the specific requirements?

Response 3.3: Thank you for this comment. We are deeply sorry for our unclear descriptions. The function of unitary matrix $\mathbf{W}$ in DNSP is to pre-encode the transmitted data. In addition, $\mathbf{W}$ is used to recover the transmitted data at the receiver. So, the unitary matrix $\mathbf{W}$ is formed by orthogonal codes, e.g., Hadamard, in [29] that satisfies the condition $\mathbf{W}^H\mathbf{W} = \mathbf{I}$. In this work, we adopt the same matrix as [12], i.e., Walsh Hadamard matrix. We have added corresponding description in the revised manuscript.

Author action: In the second sentence of the first paragraph in *System Model*, on page 4, "At the transmitter, the modulated signal $\mathbf{s} = [s_0, s_1, \ldots, s_{N-1}]^T$ is firstly precoded by an unitary matrix $\mathbf{W} \in \mathbb{R}^{N\times N}$, ..." is modified as "... an unitary matrix $\mathbf{W} \in \mathbb{R}^{N\times N}$, e.g., Walsh Hadamard matrix, to hold the orthogonality between the PS and the data sequence.".

[12] G. Dou, C. He, C. Li, and J. Gao,"Channel estimation and symbol detection for OFDM systems using data-nulling superimposed pilots," *Electron Lett.*, vol. 50, no. 3, pp. 179–180, Jan. 2014.

[29] J. C. Estrada-Jimenez and M. J. Fernandez-Getino Garcia, "Partial-data superimposed training with data precoding for OFDM systems," *IEEE Trans. Broadcast.*, vol. 65, no. 2, pp. 234–244, June 2019.

Comment 3.4: In the training phase, is the verification set generated and used to search the optimized parameters? If the validation set is adopted, the corresponding processing should be described.

Response 3.4: Thanks for your suggestion. We do agree with the reviewer that the verification set is generated and used to search the optimized parameters. We are very sorry that we did not describe verification processing clearly during network training, and have added the descriptions in the revised manuscript.

Author action: In the last sentence of the second paragraph of *Data collection* for *Model training*, on page 8, we have added the description of the validation set, i.e., “In addition, to validate the trained network parameters during the training phase, a validation set is also generated by using the same generation method of training set, and thus we can capture a set of optimized network parameters.”.

Comment 3.5: The author mentioned that after pre-training, if you change the test environment, you also need to fine-tune the network. So, what is the significance of such transfer learning?

Response 3.5: Thank you for this comment. As you think, we need to fine tune the network after changing the test environment. However, the network usually converges quickly when the fine-tuning is conducting in transfer learning. The reason is given as follows. We learn a function set (e.g., $\mathcal{F}=\{f_1, f_2, \dots, f_n\}$) rather than a function (e.g., f_n) in the pre-training phase of transfer learning [R1]. So, in the fine-tuning phase, we just need to find out which function is suitable. In addition, compared with DL-based network training, much fewer samples and shorter training time are needed in the fine-tuning phase.

Author action: In the fifth sentence of *Fine-tuning* for *Model training*, on page 10, we have added the relevant description, i.e., “In addition, compared with DL-based network training, much fewer samples and shorter training time are needed in the fine-tuning phase.”.

[R1]Y. Yang, F. Gao, Z. Zhong, B. Ai, and A. Alkhateeb, “Deep transfer learning-based downlink channel prediction for FDD massive MIMO systems,” *IEEE Trans. Commun.*, vol. 68, no. 12, pp. 7485-7497, Dec. 2020.

Comment 3.6: In the first sentence of the Definition 1 on page 4, the author gave a marginal probability distribution $P\left(h\,|h_{\mathrm{LS}}\right)$, but the h was not explained.

Response 3.6: Thanks for your careful reading. We apologize for our carelessness. We have added the corresponding descriptions in the revised manuscript.

Author action: In the first sentence of the first paragraph of *Definition 1* for *Transfer Learning*, on page 6, we have added the description of parameter h, i.e., “... , where h is the real CSI and h_{LS} is the estimated CSI by using the LS algorithm, respectively.”.

Comment 3.7: In the first sentence under Equation (1), the rounding symbol $\lfloor\cdot\rfloor$ was not explained in the Notations.

Response 3.7: Thanks for your careful reading. We apologize for our carelessness and have added corresponding explanation in the *Notations*.

Author action: In the *Notations* of the revised manuscript, we have added the explanation of the floor operation,

i.e., " $\lfloor \cdot \rfloor$ represents the floor operation.".

Reviewer 4:

Comment 4.1: I suggest removing the acronym from the title of the manuscript. Similarly, the abstract is packed with multiple acronyms which make the read challenging. To this end, the authors should move all the acronyms from the abstract to the main body of the manuscript, and should make sure that every acronym is defined exactly once at its first use.

Response 4.1: Thanks for your comments and careful reading. We have made a proofreading and corrected these acronyms.

Author action:

(1) We have removed the acronym from the title of the revised manuscript, i.e., "Deep Transfer Learning-based Channel Estimation in OFDM Systems Using Data-nulling Superimposed Pilots" has been modified as "Transfer Learning-based Channel Estimation in Orthogonal Frequency Division Multiplexing Systems Using Data-nulling Superimposed Pilots".

(2) In the revised manuscript, we have moved all the acronyms from the abstract to the main body. There are 21 acronyms, which are

Rank	Acronym	Common meaning(s)	Count
1	OFDM	Orthogonal frequency division multiplexing	36
2	CE	Channel estimation	166
3	PS	Pilot sequence	17
4	ST	Superimposed training	16
5	DNSP	Data-nulling superimposed pilot	47
6	MMSE	Minimum mean square error	44
7	DL	Deep learning	37
8	DNN	Deep neural network	7
9	CNN	Convolutional neural network	40
10	CSI	Channel state information	16
11	TL	Transfer learning	56
12	MSE	Mean square error	2
13	NMSE	Normalized mean square error	29
14	LS	Least squares	49
15	CP	Cyclic prefix	6
16	CSCG	Circularly symmetric complex Gaussian	1
17	SNR	Signal noise ratio	17
18	dB	decibe	20
19	SD	Symbol detection	35
20	BER	Bit error rate	24
21	ZF	Zero forcing	16

Comment 4.2: The description of the generated datasets, and the division into training/test samples is vague C the authors should make sure that the entire experimentation is fully reproducible and possible to understand based solely on the description provided in the manuscript. Additionally, the authors should make their data publicly available so that other research groups can reproduce the experiments (the same applies to the implementation of their deep learning model).

Response 4.2: Thanks for your comments and suggestions. We have added the information of the Dataset in *Parameter Setting* of the revised manuscript. We have added the link of the source code to the revised manuscript.

Author action:

1) From the fifth to eighth sentences in the first paragraph of *Parameter Setting*, on page 10, we have added the following sentences, i.e., "The data sets $\{\mathbb{D}_{\mathrm{S}}\}$ and $\{\mathbb{D}_{\mathrm{T}}\}$ have 8,000 and 500 samples, respectively. For both of them, the batch sizes are set as 20 samples. In pre-training phase, the data set $\{\mathbb{D}_{\mathrm{S}}\}$ is divided into training set and validation set with the sizes of 6000 and 2000, respectively. Only 500 samples are employed for the $\{\mathbb{D}_{\mathrm{T}}\}$, in which 300 and 200 samples are respectively allocated to $\{\mathbb{D}_{\mathrm{TTr}}\}$ and $\{\mathbb{D}_{\mathrm{TTe}}\}$.".

2) In the last sentence of the first paragraph in *Experimental Analysis* on page 10, we have added the footnote, i.e., "The source code is publicly available on Github[1]".

[1]The source code is available at https://github.com/Leiunnn/TransferLearningBasedCEbyDNSP.git.

Comment 4.3: The authors should confront their technique with other approaches from the literature (also, I suggest performing multi-fold cross-validation experiments to understand the abilities of the introduced techniques). Finally, the authors should back up their claims with appropriate statistical testing.

Response 4.3: Thanks for your comments and suggestions. We do agree this comment, and have added the performance comparison of the proposed work with existing technique. The added existing technique is denoted as "LS_CE [29]", "MMSE_CE [29]", "LS_CE+ZF_SD [29]", and "MMSE_CE+MMSE_SD [29]" in Fig 3 and Fig 4.

Author action:

1) In Fig 3, we have added the performance comparison of NMSE, in which we compare the CE in [12], the CE in [29], and the proposed CE. Accordingly, we have added the descriptions for Fig 3. Please see the sentences from second sentence to third sentence in the second paragraph of *CE and Symbol Detection (SD) Performance*, on page 11, i.e., "From Fig 3, the NMSE of [29] is higher than that of [12]. The reason is the partially data-dependent ST scheme is employed in [29], which superimposes the data sequence on the transmitted PS and thus introduces the superimposed interference into its CE. ...".

2) Compared with [12] and [29], the BER curves with ZF and MMSE equalizations are given in Fig 4. Correspondingly, its descriptions are added from the fourth sentence to the fifth sentence in the third paragraph of *CE and Symbol Detection (SD) Performance*, on page 13, i.e., "In Fig 4, we compare the BERs among those of [12], [29], and the proposed scheme. With the same CE methods and equalization methods, the BER of [12] is

lower than that of [29] due to the influence of CE. ...”.

3) Below equation (11) on page 11, we have modified the simplified expressions, i.e., “

- “Proposed” is used to denote the proposed TL-based CE, “No_Transfer” denotes the proposed scheme without transferring. “LS_CE [12]” and “MMSE_CE [12]” represent “LS channel estimation in [12] ” and “MMSE channel estimation in [12]”, respectively. Meanwhile, “LS_CE [29]” and “MMSE_CE [29]” represent “LS channel estimation in [29] ” and “MMSE channel estimation in [29]”, respectively.
- “LS_CE+ZF_SD [12]”, “LS_CE+MMSE_SD [12]”, “MMSE_CE+ZF_SD [12]”, and “MMSE_CE+MMSE_SD [12]” stand for the “LS channel estimation followed by zero forcing (ZF) equalization in [12]”, “LS channel estimation followed by MMSE equalization in [12]”, “MMSE channel estimation followed by ZF equalization in [12]”, and “MMSE channel estimation followed by MMSE equalization in [12]”, respectively. Meanwhile, “LS_CE+ZF_SD [29]”, “LS_CE+MMSE_SD [29]”, “MMSE_CE+ZF_SD [29]”, and “MMSE_CE+MMSE_SD [29]” stand for the “LS channel estimation followed by ZF equalization in [29]”, “LS channel estimation followed by MMSE equalization in [29]”, “MMSE channel estimation followed by ZF equalization in [29]”, and “MMSE channel estimation followed by MMSE equalization in [29]”, respectively.
- “Proposed+ZF_SD”, “Proposed+MMSE_SD”, “No_Transfer+ZF_SD”, and “No_Transfer+MMSE_SD” stand for the “proposed transfer learning channel estimation followed by ZF equalization”, “proposed transfer learning channel estimation followed by MMSE equalization”, “without transfer learning channel estimation followed by ZF equalization”, and “without transfer learning channel estimation followed by MMSE equalization”, respectively.

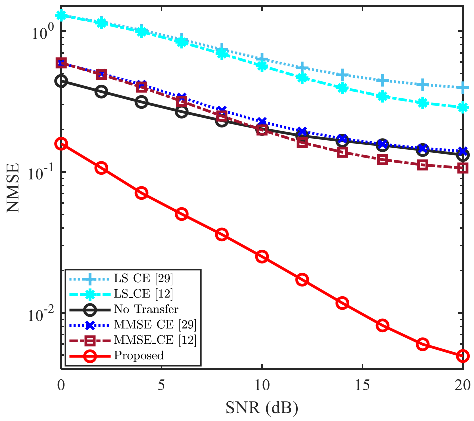

[12] G. Dou, C. He, C. Li, and J. Gao,“Channel estimation and symbol detection for OFDM systems using data-nulling superimposed pilots,” *Electron Lett.*, vol. 50, no. 3, pp. 179–180, Jan. 2014.

[29] J. C. Estrada-Jimenez and M. J. Fernandez-Getino Garcia, “Partial-data superimposed training with data precoding for OFDM systems,” *IEEE Trans. Broadcast.*, vol. 65, no. 2, pp. 234–244, June 2019.

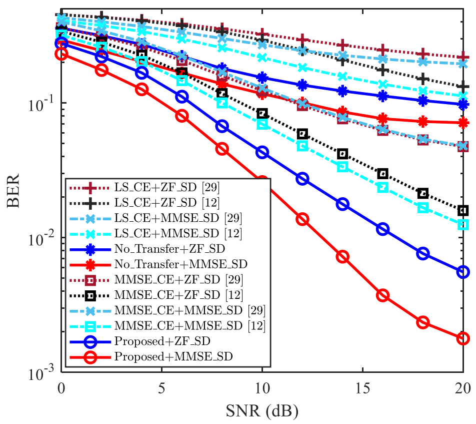

Comment 4.4: The authors should perform careful proofreading of the entire manuscript C there are quite a number of grammar issues around. Additionally, please avoid having the paragraphs that contain just one sentence. Finally, please avoid using short forms ("doesnt").

Response 4.4: Thanks for your careful reading. We have made a careful proofreading. In the revised manuscript, we have corrected the inappropriate/incorrect, tense and expression, etc.

Author action: We have made the following corrections in the revised manuscript:

1) In the first sentence of the *Transfer Learning-based Channel Estimation*, on page 5, we have corrected the "... which caused the symbol misidentification in the receiver." to "... which causes the symbol misidentification at the receiver.".
2) In the last sentence of the *Definition 3*, on page 6, we have corrected "In this paper, TL is to transfer knowledge by the ReCNN, ..." to "In this paper, TL transfers the knowledge using the ReCNN, ...".
3) In the first sentence of the *Pre-training*, on page 9, we have modified "Denote the whole network parameters as $\Theta = \{\Theta_{\text{pre}}, \Theta_{\text{fin}}\}$, with Θ_{pre} and Θ_{fin} being the sets of parameter values for *Pre-training* and *Fining-training* phase, respectively." as "We denote the whole network parameters as $\Theta = \{\Theta_{\text{pre}}, \Theta_{\text{fin}}\}$ of which the Θ_{pre} and Θ_{fin} are the sets of parameter values for *Pre-training* and *Fining-training* phase, respectively.".
4) In the fifth sentence of the *Parameter Setting*, on page 10, we have corrected the "... date sets ..." to "... data sets ...".

In addition to the corrections mentioned above, we have corrected other grammar, tense, and sentences in the revised manuscript which have been highlighted.
