# Supplementary material for: Transfer learning-based channel estimation in orthogonal frequency division multiplexing systems using data-nulling superimposed pilots
Source: PLoS One. 2022 May 27;17(5):e0268952. doi: 10.1371/journal.pone.0268952 (PMC9140250; doi:10.1371/journal.pone.0268952)
Supplement: Attachment — Submitted filename: Response to Reviewers.pdf [file pone.0268952.s011.pdf]

Revision of Manuscript ID: [PONE-D-21-33229R1] Transfer Learning-based Channel Estimation in Orthogonal Frequency Division Multiplexing Systems Using Data-nulling Superimposed Pilots

We would like to express our sincere thanks to the editor and the reviewers for their careful reviews and valuable comments, which have helped us significantly to improve the quality of our work. In the following, we address all comments in detail. All the corresponding changes are highlighted in yellow in the revised manuscript. Please assign this article to the same reviewers.

Reviewer 1:

Comment 1.1: The Revised paper has incorporated all the revisions in proper manner. And now the paper stands Accepted with no further revisions.

Response 1.1: Thank you very much for your positive comment.

Reviewer 2:

Comment 2.1: Experimental results show very good accuracy and performance Also, proposed methodology represents an improved efficiency which can be useful for society in future.

Response 2.1: Thank you very much for your positive comment.

Reviewer 4:

Comment 4.1: I am happy to see that the authors have indeed seriously addressed my concerns, and the manuscript is in better shape now. I would, however, encourage the authors to do yet another pass through the

entire paper—please avoid using vague statements in the discussion part ("performance is slightly worse"), as they should be objective. Also, it would be great to avoid having the paragraphs that contain just one sentence.

Response 4.1: Thanks for your comment and suggestion. We apologize for our vague statements in the discussion part. We have modified these statements in the revised manuscript. We do agree with the reviewer that it would be great to avoid containing just one sentence in a paragraph. Thus, we have made corresponding modifications in the revised manuscript.

Author action:

(1) We have made corresponding modifications about the vague statements in the discussion part, which are given as follows.

1) In the third sentence from back to front of the first paragraph of *CE and Symbol Detection (SD) Performance*, from page 12 to page 13, "This reflects that the "Proposed" obtains the best performance of NMSE for CE, and thus can work well under the varied environment." is modified as "For example, the NMSE of "Proposed" is 4.9×10^{-3} for the case of SNR=20dB, while the NMSE of "MMSE_CE[12]" is 1×10^{-1}. This reflects that the "Proposed" obtains higher CE accuracy than "MMSE_CE[12]", and thus it can work well in the varied environment.".
2) In the second sentence from back to front of the second paragraph of *CE and Symbol Detection (SD) Performance*, on page 13, we have added the specific BER comparison, i.e., "For the case where SNR=20dB, the BER of "Proposed+MMSE_SD" is less than 1.8×10^{-3} while the BER of "MMSE_CE+MMSE_SD[12]" is about 1.3×10^{-2}.".
3) In the first sentence of the second paragraph of *Impact of P*, on page 14, "Since the CE performance of [29] is slightly worse than that of [12], ..." is modified as "Since the "LS_CE[12]" and "MMSE_CE[12]" achieve smaller NMSEs than those of [29] with the same CE method, ...".
4) In the third sentence from back to front of the second paragraph of *Impact of P*, on page 14, we have added the specific NMSE comparison, i.e., "From Fig 5, when SNR=20dB and $P = 4$, the NMSE of "MMSE_CE[12]" is higher than 2×10^{-1}, while the NMSE of "Proposed" is lower than 3×10^{-2}.".
5) In the second sentence from back to front of the third paragraph of *Impact of P*, on page 14, we have added the specific BER comparison, i.e., "For example, for the cases where SNR=20dB and $P = 8$, the BER of "Proposed + MMSE_SD" is about 1.6×10^{-3}, while the BER of "MMSE_CE+MMSE_SD[12]" is larger than 1×10^{-2}.".
6) In the second sentence from back to front of the second paragraph of *Impact of ρ*, on page 15, we have added the specific NMSE comparison, i.e., "For example, for the cases where SNR=20dB and $\rho = 0.1$, the NMSE of "MMSE_CE[12]" is higher than 1×10^{-1}, while the NMSE of "Proposed" is lower than 1×10^{-2}.".
7) In the second sentence from back to front of the third paragraph of *Impact of ρ*, on page 16, we have added the specific BER comparison, i.e., "From Fig 8, when SNR=20dB and $\rho = 0.2$, the BER of "Proposed+MMSE_SD" is lower than 2×10^{-3}, while the BER of "MMSE_CE+MMSE_SD[12]" is about

1.2×10^{-2}.".

(2) In addition, to avoid containing just one sentence in a paragraph, we have added the following modifications in the revised manuscript.

1) In the *CE and Symbol Detection (SD) Performance*, on page 11, we have merged the first paragraph and the second paragraph into one paragraph.
2) In the fourth paragraph of *Impact of* P, on page 14, we have modified "On a whole, against the varying of P, the proposed scheme improves the performance of CE and SD, and this improvement possesses its robustness." as "On a whole, from Fig 5 and Fig 6, the proposed scheme reaches lower NMSEs and BERs than those of [12]. With the varying P, the proposed scheme still improves the performance of CE and SD, and thus possesses its robustness.".
3) In the fourth paragraph of *Impact of* ρ, on page 16, we have modified "To sum up, against the impact of ρ, the proposed scheme can effectively reduce the NMSE of CE and BER of SD, possessing its robustness." as "To sum up, the NMSEs and BERs of the proposed scheme show superiority over those of [12] in Fig 7 and Fig 8, respectively. Against the impact of ρ, the proposed scheme can effectively reduce the NMSE of CE and the BER of SD to possess its robustness.".

Besides the above comments, to improve the readability of the manuscript, we have made additional changes in the manuscript which are highlighted in yellow.
